# Phylogenomic Analyses and DNA Barcoding Development Within Moraceae: Insights Into Genomic Features, Mutational Hotspots, and Adaptive Evolution

**DOI:** 10.1002/ece3.72399

**Published:** 2025-11-09

**Authors:** Li‐Na Zhou, Shi‐Zhen Wu, Qing Ma, Xiao‐Wen Jia, Pan Li, Xin‐Jie Jin, Yong‐Hua Zhang

**Affiliations:** ^1^ College of Life and Environmental Science Wenzhou University Wenzhou China; ^2^ College of Biology and Environmental Engineering Zhejiang Shuren University Hangzhou China; ^3^ Key Laboratory of Biodiversity and Environment on the Qinghai‐Tibetan Plateau, Ministry of Education, School of Ecology and Environment Xizang University Lhasa China; ^4^ Motuo Biodiversity Observation and Research Station of Xizang Autonomous Region, Motuo China; ^5^ Zhejiang Provincial Key Laboratory of Water Ecological Environment Treatment and Resource Protection Wenzhou University Wenzhou China

**Keywords:** molecular markers, Moraceae, phylogeny, plastome, positive selection

## Abstract

Moraceae, with its seven tribes, 45 genera, and approximately 1200 species, is of significant value in food, medicine, ecological restoration, and as a source of industrial raw materials. However, determination of the taxonomy and phylogeny of Moraceae species remains challenging due to their diverse morphological features. To address this issue, we sequenced seven complete plastomes and analyzed online datasets for 46 other species at the tribal level within Moraceae. Our analysis revealed that all the plastomes within this family had a typical quadripartite structure and ranged from 158,459 to 162,594 bp in length. Comparative plastome analyses revealed 10 highly variable regions (*ndhF*‐*rpl32*, *rps4*‐*trnT*, *rps15*‐*ycf1*, *trnC*‐*petN*, *ycf1*). A total of 5350 dispersed and tandem repeats along with 4751 simple sequence repeats (SSRs) were identified, highlighting their potential for the development of molecular markers in Moraceae. While the evolutionary rates across the various tribes of Moraceae were found to be similar, the genes *ndhK*, *ndhD*, *rps2*, and *rps12* displayed evidence of positive selection. Codon usage bias was shaped by mutation and selection, with significant natural selection observed on genes such as *clpP*, *ndhC*, *ndhI*, etc. Additionally, 13 optimal codons were identified for 10 genes. This study confirms that the seven tribes within the Moraceae family are monophyletic, with divergence estimated to have occurred approximately 79.43 million years ago. This timing coincides with the formation of modern rainforests and a burst in angiosperm diversity toward the end of the Cretaceous period. Overall, the resolution of Moraceae tribal‐level phylogeny provides a foundational framework for revising taxonomic classifications. Furthermore, our genomic resources will be available for genetic engineering and germplasm exploration within this versatile plant family.

## Graphical abstract

The article presents the newly sequenced plastome maps of seven species within the Moraceae family. These species include *Broussonetia kaempteri*, *Broussonetia monoica*, *Broussonetia papyrifera* , *Malaisia scandens*, *Dorstenia contrajerva* , *Fatoua villosa* , and *Taxotrophis ilicifolia*. These plastome sequences provide valuable genomic insights for future phylogenetic and evolutionary studies within the family.

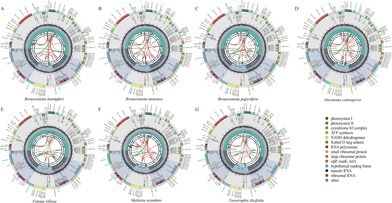

## 1 Introduction

Moraceae, which includes 7 tribes, 45 genera, and approximately 1200 species, is primarily distributed in tropical and subtropical regions, with some species also found in temperate zones (Zhang 2019; Gardner et al. 2021; Cole and Gardner 2023; Gardner 2023). The family exhibits considerable diversity and holds significant value in areas such as food, industry, medicine, and timber production (Zhang, Yang, Zhao, et al. 2022; Zhang, Yang, Li, et al. 2022). Common characteristics of this family include a latex duct system that secretes a white milky sap, spirally arranged leaves, and fruits typically being achenes or drupes (Clement and Weiblen 2009; Cardoso et al. 2018). On the other hand, the morphology of plants in the Moraceae family is highly complex, exhibiting variability in leaf shapes, inflorescence structures, fruit types, latex secretion, diverse life forms, and symbiotic relationships. This morphological complexity has posed challenges for accurate taxonomic classification within the family. From the late 19th century to the early 20th century, the classification of Moraceae became relatively stable (Berg 1978; Berg et al. 2006). Berg (2001) initially divided it into five tribes based on morphological data. Subsequently, Berg and Corner (2005) proposed a classification system consisting of seven tribes based on morphological heterogeneity, although the tribe Soroceae was not considered monophyletic. The advent of molecular phylogenetic studies has since provided new insights, leading to further taxonomic revisions. Zerega et al. (2005) confirmed the monophyly of the Moraceae based on both molecular and morphological characteristics; Clement and Weiblen (2009) reclassified Moraceae into six tribes using both morphological and molecular data. However, due to data discrepancies, Zerega et al. (2010) redefined the Artocarpeae tribe into three genera and introduced a new tribe, Paratocarpeae. Gardner et al. (2021) reinstated the classification of *Tropsis caucana* and replaced the tribe Castilleae with Antiarideae (Olmedieae) based on results from phylogenomic study. Currently, the Moraceae comprises seven tribes: Artocarpeae, Antiarideae, Dorstenieae, Ficeae, Chlorophoreae, Moreae, and Paratocarpeae. The classification of Moraceae, based on morphological and limited molecular markers, has comprehensively revealed the phylogenetic relationships among its tribes, but the precise genome‐level phylogeny of Moraceae remains uncertain. Although more recent phylogenetic research from 2021 onwards has employed advanced techniques, including direct homologous nuclear and plastomes, these studies have primarily focused on a limited number of species, particularly *Ficus*, *Morus*, and a few others within Moraceae. Consequently, these studies have been insufficient in resolving the phylogenetic relationships at the tribal level (Zhang, Yang, Zhao, et al. 2022; Zhang, Yang, Li, et al. 2022; Neo et al. 2024; Wang et al. 2024). Additionally, recent attempts have been made to differentiate species within Moraceae based on pollen morphology (Khan et al. 2025). This study, utilizing plastomes, aimed to elucidate the phylogenetic relationships at the tribal level within the Moraceae.

Building on this, recent studies have utilized plastomes and other molecular markers to further explore the phylogenetic relationships within Moraceae, yet they still face challenges in resolving tribal‐level relationships due to limited species sampling and data discrepancies. One significant limitation is the restricted sampling at the species or genus level. For instance, Neo et al.'s (2024) comprehensive study on the mulberry family (Moraceae), which included 32 plastomes from 12 genera, demonstrated length and structural variations in the cp genomes. Their findings supported existing taxonomy, although some species, such as *Malaisia scandens* and *Trophis scandens*, were incorrectly clustered with *Broussonetia* species. This suggests that while plastomes are effective for species classification, they still present challenges in resolving complex relationships, especially at deeper taxonomic levels. Moreover, the focus on genus‐level analysis has been another common approach, as exemplified by Wang et al. (2024), who examined the phylogeny of the *Morus* genus by combining plastomes and nuclear data from 13 species. Additionally, Zhang, Yang, Li, et al. (2022) focused on the genus *Ficus*, where they sequenced plastomes from 10 species and incorporated data from 46 previously published genomes. Their results indicated a conserved genomic structure, but phylogenetic analysis revealed that the traditionally recognized subgenera of *Ficus* were not monophyletic. Furthermore, the study identified divergence hotspots in intergenic spacers and genes under positive selection, such as *clpP*, *rbcL*, and *ccsA*, providing insights into the evolutionary dynamics of *Ficus*. Therefore, the current body of research reveals an overarching gap in the in‐depth exploration of plastome characteristics at the tribe level in Moraceae. While significant strides have been made in documenting plastome features within certain genera, there is a need for a more thorough analysis of the intrinsic properties of plastomes, such as structural variations and evolutionary constraints, to better understand their role in shaping plant phylogenies at the tribe level.

In light of these challenges, this study focuses on three key objectives: (1) conducting a comparative analysis of plastome features across various tribes and species within the Moraceae to develop efficient DNA barcodes for species identification; (2) constructing tribe‐level phylogenetic trees based on plastomes and estimating divergence times for each tribe; (3) uncovering the evolutionary mechanisms of different tribes and major evolutionary branches within Moraceae, including the identification of molecular markers, analysis of codon usage bias, and assessment of natural selection pressures. Based on the availability of chloroplast genome data in the NCBI database, species representativeness, and practical accessibility of samples for the seven tribes within the Moraceae family, we intensified sampling for the tribe Dorstenieae. This included three species of *Broussonetia* (which serve as vital resources for papermaking and traditional medicine in Asia), *Malaisia scandens*, *Dorstenia contrajerva* , and *Fatoua villosa* . Additionally, a sample of *Taxotrophis ilicifolia* representing the basal lineage *Taxotrophis* within the tribe Moreae was included. Through these objectives and samples, the study aims to deepen our understanding of the evolutionary history and phylogenetic relationships of Moraceae plants, and to reveal potential adaptive evolutionary patterns. Specifically, the resolution of Moraceae tribal‐level phylogeny provides a foundational framework for revising taxonomic classifications, offering crucial scientific support for species conservation, resource utilization, and systematic research.

## 2 Materials and Methods

### 2.1 Plant Materials, DNA Extraction, and Genome Sequencing

For this research, we collected leaf samples from seven Moraceae species for genome sequencing (species in bold; Table S1). We extracted genomic DNA from silica‐dried leaf tissue using an optimized cetyltrimethylammonium bromide (CTAB) protocol and evaluated the DNA quality and quantity using agarose gel electrophoresis.

Paired‐end libraries (150‐bp read length) were prepared from each species, indexed with unique barcodes, and pooled. This pooled library was sequenced on one lane of an Illumina HiSeq 2500 at the Beijing Genomics Institute (BGI, Shenzhen, China) for genome sequencing. We purified the sequencing data using fastp v.0.23.1 (Chen et al. 2018), removing reads: (1) containing > 10% undefined bases (N); (2) where > 50% of bases had low quality (*Q* ≤ 5); and (3) containing adapter sequences. This yielded a minimum of 6 Gbp of high‐quality, paired‐end clean reads per sample, enabling downstream analysis.

### 2.2 Plastome Assembly and Annotation

For the newly sequenced Moraceae species, we employed the GetOrganelle pipeline (Jin et al. 2020) for de novo assembly of the complete circular plastomes. The genomes were annotated automatically using CPGAVAS2 (Shi et al. 2019), followed by adjustments made with Geneious v. 2021.2.2 (Kearse et al. 2012) based on the *Broussonetia papyrifera* reference plastome (accession number: NC_035569). Circular plastome maps were drawn using OrganellarGenome DRAW (Lohse et al. 2007). Additionally, we downloaded plastomes of all Moraceae tribes from the National Center for Biotechnology Information (NCBI) database and the National Genomics Data Center's project PRJCA002187. A consistent methodology from this study's research approach was used to reannotate a total of 48 previously published genomes. The species included two outgroup species ( *Boehmeria nivea* and *Cannabis sativa* ) and 46 members of the Moraceae family: 13 Moreae species, two Antiarideae species, three Dorstenieae species, one Paratocarpeae species, 17 Ficeae species, two Chlorophoreae species, and eight Artocarpeae species. Detailed information is provided in Tables S1 and S2.

To identify the unique characters of the Moraceae plastomes, we used Geneious v. 2021.2.2 to compare GC content, genome size, and noncoding region among the 53 plastomes of Moraceae and two outgroups.

### 2.3 Plastomes Structure and Comparative Genomics Analysis

In our comparative genomics study, we aligned the plastomes of 53 (7 newly determined taxa + 46 public taxa) Moraceae species using mVISTA (Frazer et al. 2004) with LAGAN mode (Brudno et al. 2003), which allowed for the visualization of conserved and diverse sequence regions. *Afromorus mesozygia* served as the reference sequence, ensuring that the data conformed to the specific format required for effective alignment visualization on mVISTA. We then probed the IR boundaries of these genomes using IRscope (Amiryousefi et al. 2018), which allowed us to monitor changes at the junction sites of the IR, LSC, and SSC regions, with a focus on the dynamics of IR expansion and contraction as well as gene boundary shifts.

Repetitive sequences, which are DNA fragments that recur within the genome, account for a significant portion of its length. These sequences can be categorized based on their distinct characteristics and origins into several types. The three main types we focus on are: (1) Simple sequence repeats (SSRs), also known as microsatellites, usually comprising 2–6 nucleotide units per repeat. These categories reflect the diverse nature and function of repetitive sequences in genomic organization and evolution. (2) Transposable elements (TEs), also known as transposons, which are DNA fragments capable of moving within the genome. These include DNA transposons and retrotransposons, typically dispersed throughout the genome, generally short in length, and low in copy number, scattered rather than continuous. (3) Tandem repeats, also known as satellite DNA, which consist of consecutive repetitions of short DNA sequence units within the genome. In our study, we identified microsatellites using the MISA‐web tool (Beier et al. 2017), employing specific parameters to detect nucleotide repeats of various sizes. We defined the minimum number of repeats for mononucleotide motifs at 10, dinucleotide motifs at 5, trinucleotide motifs at 4, and for tetranucleotide, pentanucleotide, and hexanucleotide motifs, the threshold was set at 3 repeats. Compound microsatellites were categorized as two microsatellites located within 100 base pairs of each other. To identify dispersed and tandem repetitive sequences, we utilized REPuter (Kurtz et al. 2001) and Tandem Repeats Finder (Benson 1999) respectively, setting parameters to identify repeats of significant size and homology. Subsequently, we engaged Mafft (Katoh and Standley 2013) to assemble a plastome matrix from the 53 Moraceae samples, which was then inputted into DnaSP v5.0 (Librado and Rozas 2009) to analyze nucleotide polymorphism with predetermined window and step lengths.

To identify single nucleotide variants (SNVs) and insertions/deletions (Indels), a specialized SNV/Indel script (Chen et al. 2020) was applied, followed by visualization using R's ggplot2 package (Wickham 2016), with *Boehmeria nivea* serving as the reference sequence. This helped in detecting SNVs and Indels both across the 53 sequences and within each tribe. The genetic distances between these plastomes were calculated after aligning protein‐coding genes using Mafft and then analyzed in Mega X (Kumar et al. 2018), resulting in a comprehensive .xlsx file of the genetic distances. These data were ultimately visualized in a heatmap generated by TBtools (Chen et al. 2020), providing a clear representation of genetic relationships within the family.

### 2.4 Phylogenetic Analysis

In our research, phylogenetic trees were generated employing both maximum likelihood (ML) and Bayesian inference (BI) approaches, with outgroups designated as *B. nivea* (NC_056894) from Urticaceae and *C. sativa* (KP274871) from Cannabaceae. For the construction of phylogenetic trees utilizing the complete set of 55 (7 new + 46 published taxa, plus 2 outgroups) plastomes, one of the duplicated inverted repeat (IR) regions was removed; meanwhile, for trees constructed from protein‐coding gene sequences, duplicate protein‐coding gene sequences were excluded. The ML tree construction utilized IQ‐TREE 2.0.5 (Minh et al. 2020), applying the GTR + I + G model with 1000 iterations for ultrafast bootstrap approximation (UFBoot) and SH‐aLRT tests, with CPU core count set automatically. The command line for this process was “iqtree2 ‐s 55.fasta ‐m GTR+I+G ‐b 1000 ‐alrt 1000 ‐nt AUTO.” In the IQ‐TREE analysis, the best‐fit model was determined using its built‐in ModelFinder algorithm. Bayesian tree construction was facilitated by CIPRES (Miller et al. 2015), initially determining the optimal model and parameters with ModelTest v3.7 (Drummond et al. 2002), choosing GTR + I + G, and running for 1 × 10^6^ generations with samples every 1000 generations. The best‐fit nucleotide substitution model (Cprev) was selected based on three measures (the Akaike information criterion [AIC], the corrected Akaike information criterion [AICc], and the Bayesian information criterion [BIC]) in ModelTest v3.7 (Drummond et al. 2002). These parameters were then incorporated into the nexus matrix file for Bayesian analysis with the command “prset statefreqpr = dirichlet(1,1,1,1); lset applyto = (all) nst = 6 rates = invgamma;”. This comprehensive approach to phylogenetic tree construction underscores the rigor of our methodology.

### 2.5 Molecular Dating

We analyzed plastomes from 53 Moraceae species, selecting *B. nivea* (NC_056894) from Urticaceae and *C. sativa* (KP274871) from Cannabaceae as outgroups. The scarcity of Moraceae fossil records necessitated the use of four fossil calibration nodes and one secondary calibration node for estimating the divergence times of Moraceae species. We selected fossil nodes based on the criteria established by Zhang (2019). Zhang (2019) conducted rigorous phylogenetic placement analyses for each fossil: they accurately determined the fossil's placement node in the evolutionary tree by referring to the original fossil descriptions, subsequent research comments, and the latest phylogenetic trees of each family. Meanwhile, they strictly revised the absolute age or geological age range of each fossil with reference to the latest stratigraphic and geological time scales published by the International Commission on Stratigraphy to ensure the accuracy of time constraints. Based on this, we selected 4 Moraceae fossils as calibration nodes, namely: the root node of *Broussonetia* (33.9 MYA; Collinson 1989), the root node of *Ficus* (64 MYA; Collinson 1989), the root node of *Morus* (33.9 MYA; Collinson 1989), and the root node of *Artocarpus* (56 MYA; Hooper et al. 2010), all adopting minimum age constraints. Since most fossils can typically only provide the minimum age of the clade to which they can be safely attributed, all fossil calibrations used a uniform prior distribution, with the upper (younger) boundary of the age range of the oldest stratum where the fossil is located serving as the minimum limit. The secondary calibration node, marking the divergence between Moraceae and Cannabaceae, was positioned at the crown node at 91 MYA (Zhang 2019). This calibration value is derived from the work of Zhang (2019), who constructed a deep divergence time framework for Rosales through fossil calibrations across multiple families of the order (including reliable fossils of Cannabaceae‐related groups). Within this framework, the crown group divergence time between Moraceae and Cannabaceae was estimated to be 91 MYA. This result has been validated by multiple methods such as Bayesian molecular clock models, demonstrating high reliability. Given that Moraceae and Cannabaceae are sister groups in phylogeny, this node can serve as a “deep‐time anchor” for the divergence of inner groups within Moraceae, supplementing the deficiency of fossil calibrations at early nodes.

A Bayesian evolutionary tree, incorporating divergence times and topology, was constructed using BEAST v1.8.0 (Drummond and Rambaut 2007) through a Markov chain Monte Carlo (MCMC) method, based on the fixed positions of the outgroup and four genera. To lessen computational demands, shared genes among species were identified, and their Pi values calculated using DnaSP v5.0 (Librado and Rozas 2009), categorizing them into high (> 0.02), medium (0.01–0.02), and low (< 0.01) variability regions. Sequences from these regions were concatenated into three files and exported in fasta format using Geneious v. 2021.2.2. These files were then merged into a single nexus format file with phyloSuite v1.2.3 (Xiang et al. 2023), partitioned, and parameters set in BEAUTi (Drummond et al. 2012). The analysis involved setting a joint tree for partitions; taxa settings for *Broussonetia*, *Artocarpus*, *Ficus*, *Morus*, and Cannabaceae with divergence times using a Normal prior distribution with a variance of 1.0 Ma; an uncorrelated relaxed clock model for molecular clock modeling; a Yule Model for tree priors; and the selection of the GTR + I + G model for each partition using ModelTest. The MCMC chain was run for 800 million iterations, sampling every 1000th chain. After configuring all parameters, the XML file was processed with BEAST v1.8.0. The MCMC's convergence was verified with Tracer v1.7.1 (Rambaut et al. 2018), where an Effective Sample Size (ESS) > 200 suggested a reliable topology. The initial 20% of samples were discarded using Tree Annotator v1.10.4, and the final tree file, annotated with divergence times, was visualized and refined with Figtree v1.4.4.

### 2.6 Codon Usage Bias Analysis

Geneious Prime 2021 was used to extract protein‐coding gene sequences from 53 plastomes, selecting sequences postduplicate removal, with a focus on those at least 300 bp long and divisible by three. Codon usage bias was assessed by merging genes into a single sequence and analyzing with CodonW v.1.4.4 (https://codonw.sourceforge.net/), which evaluated parameters including the effective number of codons (ENC), GC content, and relative synonymous codon usage (RSCU). Each gene's codon usage bias was calculated individually, employing a matrix‐based method to organize genes by family and clade, facilitating ENC‐plot and PR2‐plot analyses. The ENC‐plot utilized the equation ENC = 2 + GC_3_ + 29/GC_3_^2^ + (1 − GC_3_)^2^ to contrast observed ENC values with expected ones. The PR2‐plot visually represented the nucleotide composition at the third codon position. Genes were ranked by ENC values to pinpoint optimal codons, analyzing the highest and lowest 5% for RSCU values. The ΔRSCU formula, ΔRSCU = RSCU_high expression_ − RSCU_low expression_, determined optimal codons with ΔRSCU ≥ 0.08 and ΔRSCU > 1, highlighting codons with both high frequency and expression as optimal.

### 2.7 Analysis of Selective Pressure

Geneious Prime v2021 was utilized to extract common protein‐coding sequences (CDS) from 53 Moraceae plastome sequences, forming sequence matrices for the 79 shared genes, each treated as a unit. The exported fasta format from Geneious Prime v2021 was converted into the fas format for upload to DnaSP v5.0. Using DnaSP v5.0, with *B. nivea* (Urticaceae) serving as the outgroup, pairwise synonymous (*Ks*) and nonsynonymous (*Ka*) substitution rates for the protein‐coding sequences were calculated at both the species and gene levels. Excel was employed to compute the ratio of nonsynonymous to synonymous substitution rates (*Ka/Ks*, *ω*), with the resulting values used to categorize genes by function and to analyze selective evolutionary pressures within different Moraceae tribes and evolutionary branches.

## 3 Results

### 3.1 Structure and Characteristics of Plastomes in Moraceae

**FIGURE 1:**
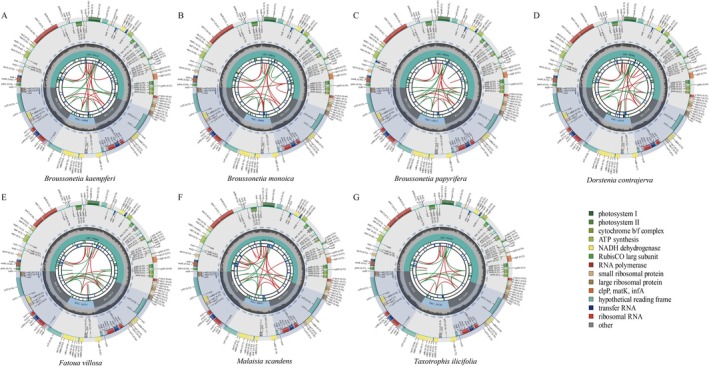
Seven newly sequenced plastome maps. (A) *Broussonetia kaempteri*; (B) *Broussonetia monoica*; (C) *Broussonetia papyrifera* ; (D) *Malaisia scandens*; (E) *Dorstenia contrajerva* ; (F) *Fatoua villosa* ; (G) *Taxotrophis ilicifolia*.

**FIGURE 2:**
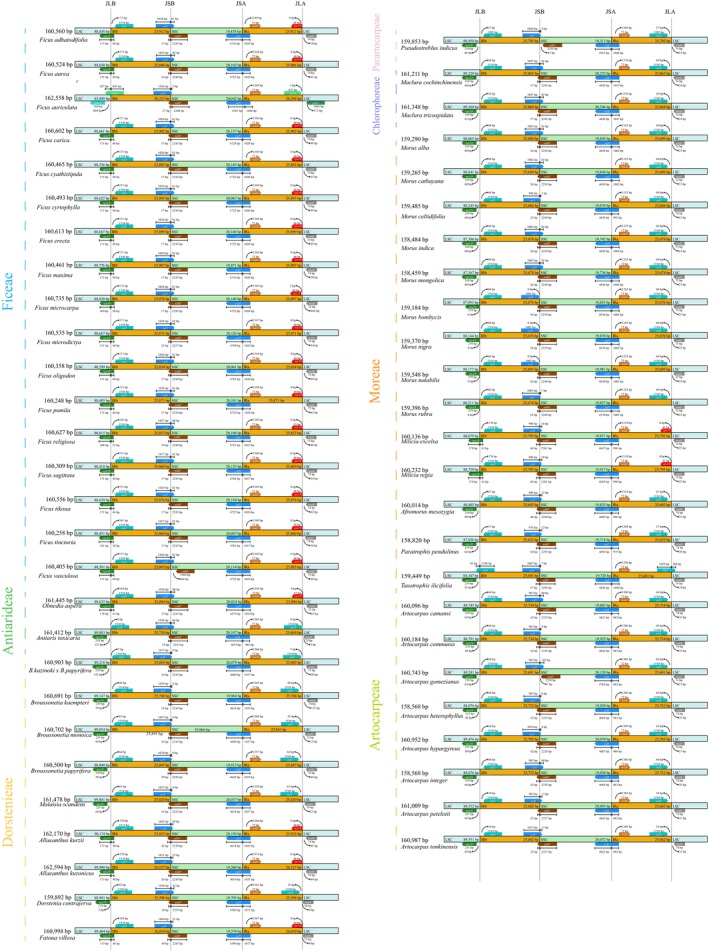
Analysis of IR boundary expansion and contraction in plastomes of 53 Moraceae species.

The plastomes of seven newly sequenced Moraceae species were assembled, displaying a typical quadripartite structure. These genomes ranged in size from 159,449 to 161,478 bp and included 135–137 genes, comprising protein‐coding genes, tRNA genes, rRNA genes, and pseudogenes (Figure 1, Tables S2 and S3). An analysis of 53 Moraceae plastomes revealed sizes between 158,459 and 162,594 bp, with approximately 136 genes, including 88–91 protein‐coding genes, 37 tRNA genes, 8 rRNA genes, and 3 pseudogenes (Tables S3 and S4). Notably, *Broussonetia kaempferi*, *B. monoica*, and *B. kazinoki* × *B. papyrifera* lacked the *rpl22* gene, reducing their protein‐coding gene count to 87. Conversely, the *rpl22* gene terminated prematurely in *Malaisia scandens*, *Allaeanthus kurzii*, *A. luzonicus* , *Dorstenia contrajerva* , and *Fatoua villosa* (Figure 1). Furthermore, the *ycf15* gene showed premature termination in *Milicia excelsa* , *M. regia* , *Paratrophis pendulinus*, *Afromorus mesozygia*, *Taxotrophis ilicifolia*, and *F. villosa* (Figure 2). In most Moraceae species, the *rps19* gene spanned the boundary between the large single‐copy (LSC) region and the inverted repeat (IRb) region, resulting in the formation of a *Ψrps19* pseudogene in the IRa region (Figure 2). Uniquely, the plastome of *Ficus auriculata* included 139 genes due to an expansion of the IR regions (Figures S1 and S2, Tables S3 and S4). The plastome of *Dorstenia contrajerva* exhibited the lowest overall GC content within the family, ranging from 35.3% to 36.4%. It showed the lowest values in the LSC region (32.6%), the SSC region (27.9%), and the protein‐coding regions (37.1%; Table S5). In contrast, *Morus indica* displayed the highest GC content in total, LSC (34.1%), and IR regions, underscoring significant differences compared with other Moraceae species (Table S5). Additionally, the IR regions generally had higher GC content than the LSC and SSC regions, with *D. contrajerva* 's IR region reaching 42.9% GC, while the SSC region showed the lowest GC content (Table S5).

**FIGURE 3:**
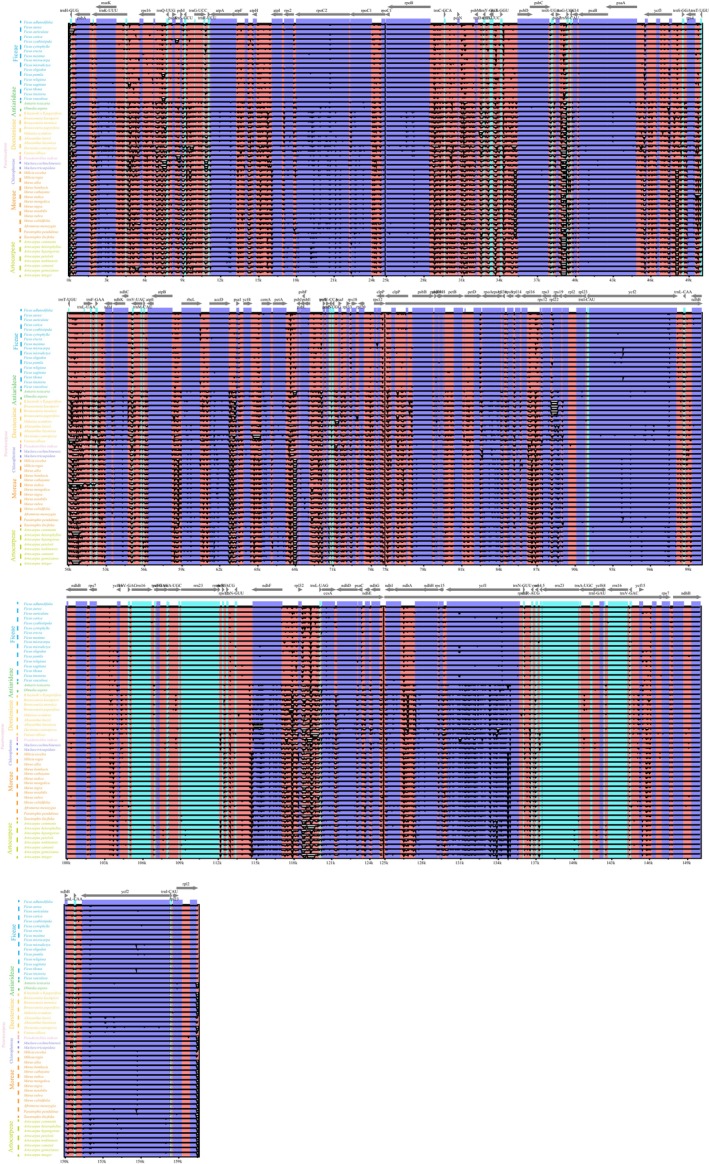
Comparative analysis of the plasomes from 53 species within the Moraceae family was performed using the mVISTA software. In the resulting alignment, gray arrows indicate the names and transcription directions of genes. Exonic regions are marked in purple, intronic regions in light blue, and noncoding sequences in pink.

Plastomes exist as circular quadripartite structures, where the boundaries—JLA (Junction IRa‐LSC), JLB (Junction LSC‐IRb), JSA (Junction SSC‐IRa), and JSB (Junction IRb‐SSC). In the studied 53 Moraceae species, multiple genes and pseudogenes such as *rps19*, *rpl2*, *rpl16*, *Ψycf1*, *ndhF*, *ycf1*, and *trnN* were located at these boundaries (Figures S1 and S2). Specifically, the *rps19* gene spanned the JLB boundary in 17 species of Ficeae, two species of *Milicia* in the Moreae, two species of *Allaeanthus* in Dorstenieae, and *Olmedia aspera* in Antiarideae, with a spanning range of 33–48 bp (Figures S1 and S2). The presence of the *Ψrps19* pseudogene in the IRa region corresponded with the *rps19* gene spanning the JLB boundary, due to the conservation of the inverted repeat (IR) areas. The *Ψycf1* pseudogene typically spanned the JSB boundary. The *ndhF* gene spanned the JSB boundary in 46 out of 49 Moraceae species, except in *Pseudostreblus indicus*, *Artocarpus gomezianus*, and *F. vasculosa* . Furthermore, sequence overlap occured between *Ψycf1* and *ndhF* in the SSC region, *rpl16* was located at the IR boundary due to the expansion in *F. auriculata* , and the *ycf1* gene extensively spans the JSA boundary with a range of 952–1651 bp. The *rpl2* gene was unique in *Taxotrophis ilicifolia* as it spanned from the JLA boundary into the LSC region. These boundary changes and gene localizations reflected the complexity and evolutionary dynamics of the plastomes of Moraceae. Comparative genomic analysis further showed that coding regions were highly conserved among closely related species, whereas noncoding regions exhibited greater variability, particularly in the LSC and SSC areas compared with the IR regions. This finding was supported by visual analyses using the mVISTA software (Figure 3), highlighting the complexity and diversity of plastomes in evolution.

### 3.2 Analysis of Plastome Variation

**FIGURE 4:**
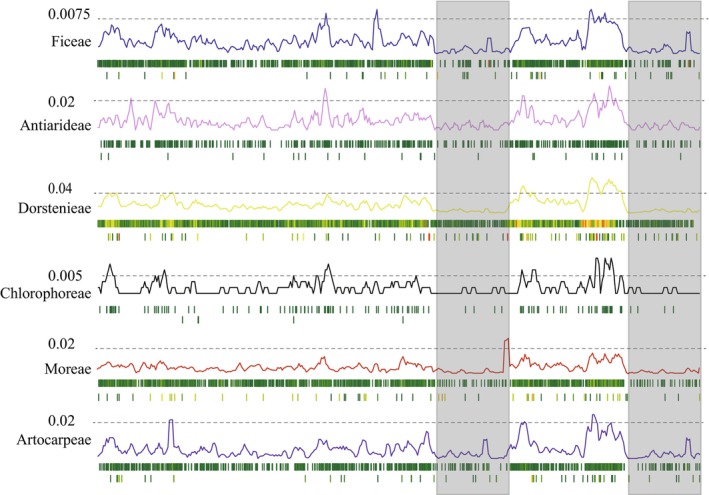
Analysis of nucleotide polymorphism and insertion/deletion across the seven Moraceae tribes. The curve represents nucleotide polymorphism, with dashed lines indicating positions of relatively high Pi (π) values. The boxes beneath the curve show the distribution of single nucleotide variants (SNVs) at the top and insertions/deletions (Indels) at the bottom. The gray shaded areas denote the approximate extent of the inverted repeat (IR) regions.

Nucleotide polymorphism and SNV/Indel analyses were conducted across different tribes of the Moraceae family, with genome variation sites quantified per 0.1 kb. Initially, comparisons across different regions of the plastome revealed higher nucleotide diversity and mutation frequency in the SSC region, while the IR regions exhibited more conservative characteristics (Figure 4). Subsequent comparisons among different Moraceae tribes showed consistent trends in nucleotide diversity and mutation distribution, with most SNVs and Indels concentrated in the SSC region and fewer variation events in the IR regions (Figure 4).

**FIGURE 5:**
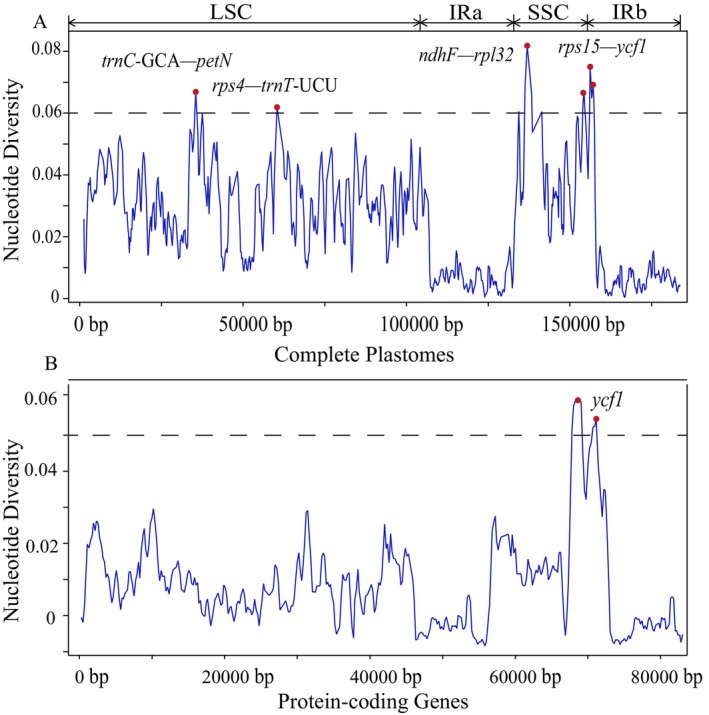
Nucleotide polymorphism analysis of plastomes in 53 Moraceae Species. This figure provides a comprehensive analysis of nucleotide polymorphism, encompassing both the entire plastome and specifically within the protein‐coding genes of plastid genomes across 53 Moraceae species.

In an analysis of nucleotide polymorphism across 53 Moraceae plastomes, four regions with Pi values greater than 0.06 were identified as high variability areas: *trnC‐GCA*–*petN*, *rps4*–*trnT‐UCU*, *ndhF*–*rpl32*, and *rps15*–*ycf1*. The first three were located in noncoding regions, while *rps15*–*ycf1* was within a coding region. This suggests that polymorphisms predominantly occurred in intergenic spacers, where Pi values were significantly higher than in coding regions. Additionally, the results indicated that nucleotide polymorphism in the IR regions was markedly lower than in the SSC and LSC regions (Figure 5A). Further analysis of nucleotide polymorphism within the protein‐coding regions extracted from the same 53 Moraceae plastomes revealed that one gene, *ycf1*, located in the SSC, had a Pi value greater than 0.06. Consistent with previous findings, nucleotide polymorphism was much higher in the SSC region compared with the LSC and IR regions, in the order SSC > LSC > IR (Figure 5B).

To validate the effectiveness of highly variable regions and genes as DNA barcodes, we constructed maximum likelihood (ML) phylogenetic trees based on the spacer regions *trnC‐*GCA*–petN*, *rps4–trnT‐*UCU, *ndhF–rpl32*, *rps15–ycf1*, and the gene region *ycf1* (Figures S3–S7). The results revealed that at the tribal level, the *ycf1* gene exhibited the highest discriminatory power, achieving 100% support for all tribes except Dorstenieae (99%). The *trnC‐*GCA*–petN* spacer ranked second, with 99% support for Dorstenieae, 97% for Moreae, and 100% for other tribes. Both regions perfectly resolved all seven tribes as monophyletic clades. In contrast, the performance of the remaining three regions was comparatively weaker. Although they failed to resolve all seven tribes into monophyletic groups, the discriminatory power of the *rps4–trnT‐*UCU region surpassed that of *rps15–ycf1*, while *ndhF–rpl32* performed the poorest among them. At the species level, only the *ycf1*‐based phylogeny demonstrated excellent resolution, with topological structures consistent with the plastome‐based phylogenetic tree for all tribes except Moreae and Ficeae. However, the other four regions exhibited misclassifications in phylogenetic relationships.

**FIGURE 6:**
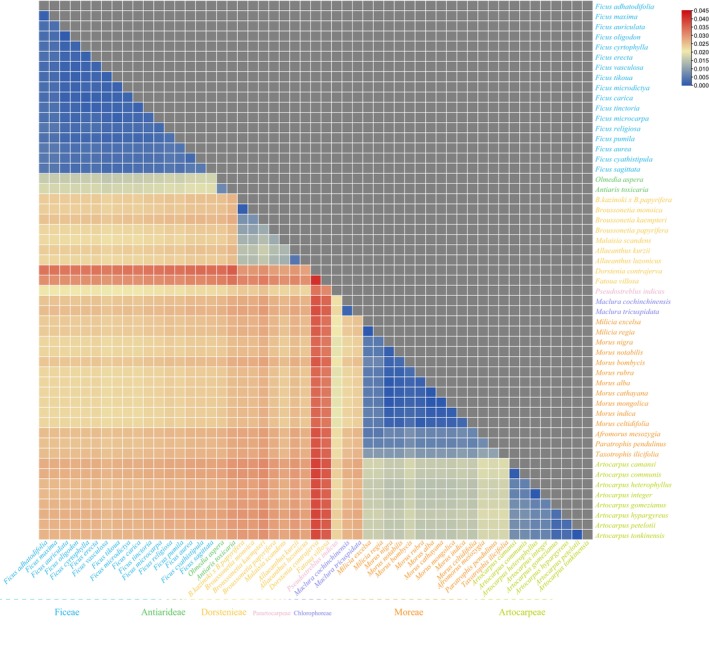
Heatmap of genetic distances among the plastid genomes of 53 Moraceae species.

Genetic distances among 53 Moraceae species were calculated using MEGA X software, a tool that measures the degree of genetic difference between species or individuals based on the number of allelic substitutions per site. The range of genetic distances among the 53 Moraceae species varied from 0 to 0.4, indicating varying degrees of genetic diversity. The largest genetic distance, 0.04, was observed between *F. villosa* and *D. contrajerva* . Conversely, the smallest genetic distance, 0, was noted between *A. integer* and *A. heterophyllus* , as well as between *A. petelotii* and *A. tonkinensis* (Figure 6).

### 3.3 Repetitive Sequences in Moraceae Plastomes

**FIGURE 7:**
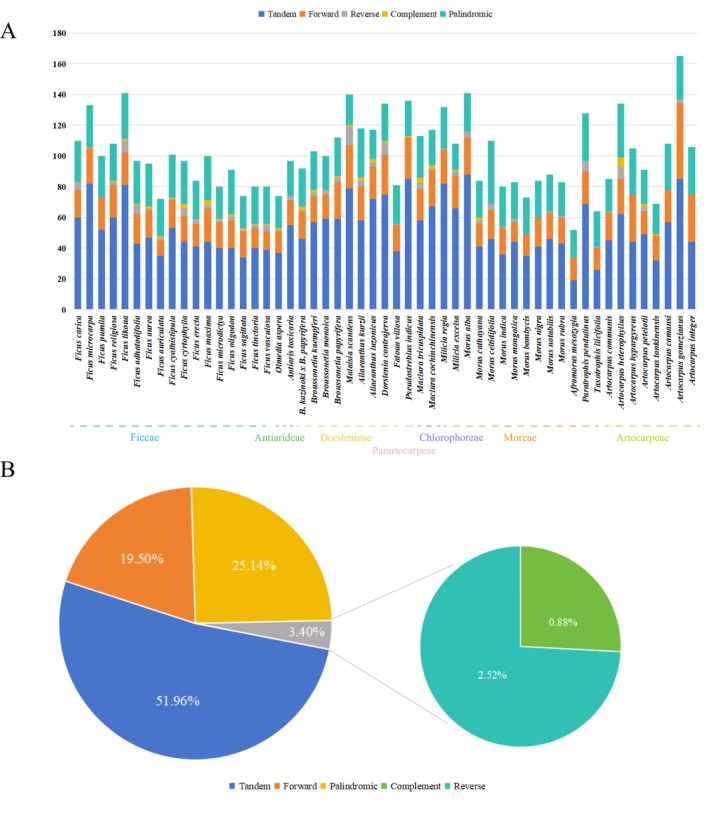
Information statistics of tandem repeat sequences and dispersed repeat sequences of 53 Moraceae species. (A) Total number of tandem repeat sequences and dispersed repeat sequences in each sample. (B) Proportional statistics of tandem repeat sequences and dispersed repeat sequences.

Using the REPuter software, scattered repeat sequences were detected across the plastomes of 53 Moraceae species, excluding the two intrinsic inverted repeat regions (IRs), the largest palindromic repeats in the plastome. Four types of transposable elements (TEs) were identified: forward repeats (F), reverse repeats (R), complement repeats (C), and other forms of palindromic repeats (P). In total, 1043 forward repeats, 1345 palindromic repeats, 135 reverse repeats, and 47 complement repeats were detected (Figure 7). *A. gomezianus* contained the highest number of forward repeats (49), while *F. auriculata* had the fewest (10). *M. celtidifolia* had the most palindromic repeats (41), and *M. excelsa* the fewest (17). Reverse and complement repeats were comparatively rare. *M. scandens* had the most reverse repeats (12), while *A. mesozygia*, *P. indicus* , *F. pumila* , *A. hypargyreus*, *A. tonkinensis* , *A. camansi*, and *A. integer* had none. *A. heterophyllus* had the most complement repeats (6), whereas 24 other Moraceae species had none, and 18 species had only one. Seven species had two complement repeats, and *F. cyrtophylla*, *F. maxima* , and *M. tricuspidata* each had three (Figure 7; Table S6).

Using the Tandem Repeats Finder software, a total of 2780 tandem repeats were identified in the plastomes of 53 Moraceae species (Figure 7). *M. alba* contained the highest number of tandem repeats with 88, while *A. mesozygia* had the fewest at 19, representing the lowest count among all examined Moraceae species. Overall, tandem repeats were more prevalent than scattered repeat sequences among the 53 Moraceae species, comprising 51.96% of the total repetitive sequences (Figure 7B). Scattered repeat sequences were primarily concentrated in intergenic spacer (IGS) regions and within introns of some protein‐coding sequences.

**FIGURE 8:**
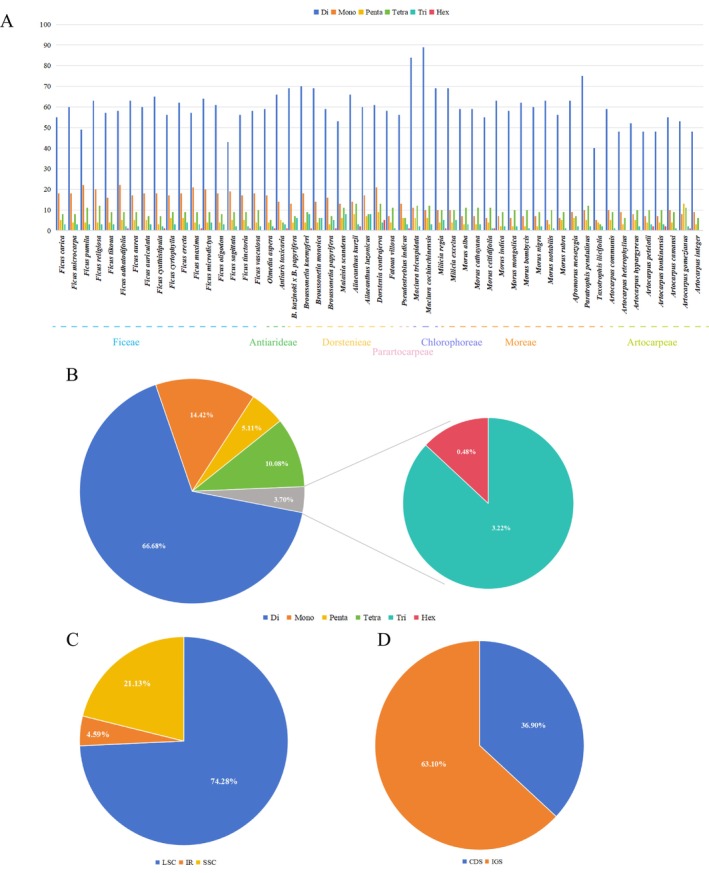
Information statistics of simple repeat sequences of 53 Moraceae species. (A) Distribution of six types of simple repeat sequences in each sample. (B) Proportion of six types of simple repeat sequences. (C) Proportion of simple repeat sequences in the LSC region, IR region, and SSC region. (D) Proportion of simple repeat sequences in protein‐coding regions (CDS) and noncoding regions (IGS).

Using MISA‐web, an analysis of simple sequence repeats (SSRs) was conducted on the plastomes of 53 Moraceae species, identifying a total of 4751 SSRs, ranging from mono‐ to hexanucleotide repeats. *M. cochinchinensis* had the highest number of SSRs with 120, while *T. ilicifolia* had the fewest at 54 (Figure 8A). Among the six types of repeats, mononucleotide repeats were the most common, totaling 3168 and accounting for 66.68% of all identified SSRs. The other types were distributed as follows: 685 dinucleotide repeats (14.42%), 243 trinucleotide repeats (5.11%), 479 tetranucleotide repeats (10.08%), 153 pentanucleotide repeats (3.22%), and the least common, hexanucleotide repeats, with only 23 instances (0.48%), found in 16 Moraceae species, with *D. contrajerva* having the most (5) (Figure 8B). The majority of SSRs were located in the large single‐copy (LSC) region of the plastome, with 3529 repeats constituting 74.28% of all SSRs. The SSC region contained 1004 SSRs (21.13%), and the IR regions had 218 (4.59%; Figure 8C). Additionally, SSRs were predominantly found in intergenic spacer (IGS) regions and within introns of some protein‐coding sequences, with 2998 SSRs located in the IGS regions (63.10%) and 1753 in protein‐coding areas (36.90%; Figure 8D; Table S7).

### 3.4 Phylogenetic Analysis and Divergence Time Estimation

**FIGURE 9:**
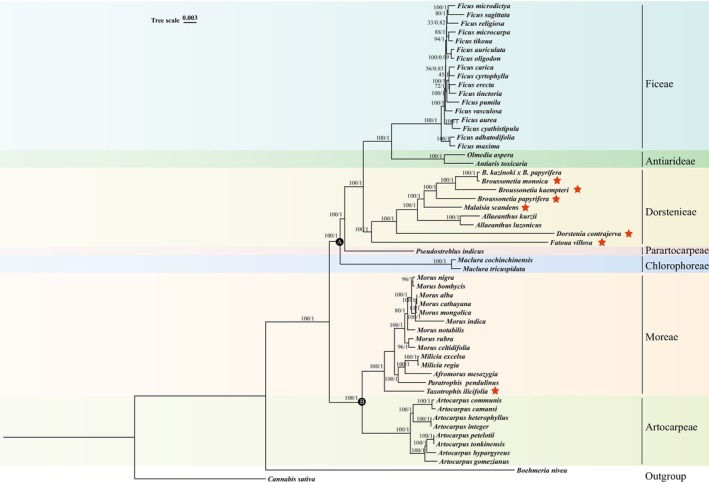
Phylogenetic tree based on plastomes of 53 Moraceae species. This tree was analyzed using maximum likelihood (ML) and Bayesian inference (BI) methods, with nodes A and B representing two major evolutionary clades. The seven species sequenced in this study are indicated by red pentagrams.

*Boehmeria nivea* from the Urticaceae and *C. sativa* from the Cannabaceae were selected as outgroups. Two datasets were utilized for sequence matrix analysis (Table S8): one dataset included the LSC, SSC, and IR with an aligned matrix length of 161,067 bp, containing 19,443 (12.07%) parsimony‐informative sites, 19,156 (11.89%) singleton sites, and 122,468 (76.04%) constant sites; the second dataset comprised protein‐coding genes (excluding duplicated genes) with an aligned matrix length of 73,122 bp, containing 6436 (8.80%) parsimony‐informative sites, 6402 (8.76%) singleton sites, and 60,284 (82.44%) constant sites. Phylogenetic results (Figures 9, S8 and S9) from both datasets and methods consistently supported the monophyly of seven Moraceae tribes and effectively differentiated the family into two evolutionary clades, Clade A and Clade B. Clade A included the tribes Dorstenieae, Ficeae, Antiarideae, Parartocarpeae, and Chlorophoreae, while Clade B comprised the tribes Artocarpeae and Moreae. For the newly sequenced seven species, all four phylogenetic trees showed consistent topology, with *B. monoica* and a hybrid of *B. kazinoki* × *B. papyrifera* forming a sister relationship, and *M. scandens* and the genus *Broussonetia* clustering as sister branches. *D. contrajerva* and the genus *Fatoua* each formed monophyletic groups within the tribe Dorstenieae, while *T. ilicifolia*, belonging to the tribe Moreae, and *P. pendulinus* from the genus *Paratrophis* clustered as sister taxa. In phylogenetic trees constructed from full‐length sequences, the topology remained consistent across all species. However, in trees based on coding sequences (CDS), inconsistencies were observed within the Ficeae, while the topology for other species remained stable. For example, *F. carica* , *F. cyrtophylla*, *F. erecta* , *F. macrocarpa* , *F. religiosa* , *F. sagittata*, *F. tikoua*, and *F. tinctoria* within the Ficeae not only exhibited inconsistent topologies but also displayed low support values. Apart from the Ficeae, the relationship between *A. hypargyreus* and *A. gomezianus* differed across the two datasets: in the CDS matrix, *A. hypargyreus* was closest to *A. tonkinensis* , while in the full‐length matrix, *A. gomezianus* was closest to *A. tonkinensis* .

The phylogenetic trees constructed exhibited variable support levels across different nodes. For the trees derived from the CDS matrix (Figures S8 and S9), the branch comprising *M. alba* , *M. cathayana*, and *M. mongolica* had a maximum likelihood (ML) support rate of 91, while the Bayesian inference (BI) posterior probability was 1. The branch including *A. petelotii*, *A. tonkinensis* , and *A. gomezianus* received an ML support rate of 84 and a BI posterior probability of 1. In contrast, for the phylogenetic trees built from the full‐length sequence (excluding duplicated regions) matrix, the same *Morus* branch had an ML support of 81 and a BI posterior probability of 1, while the *Artocarpus* branch maintained an ML support of 100 and a BI posterior probability of 1. Within the Ficeae tribe, several nodes across both sequence matrices showed lower support and posterior probabilities.

**FIGURE 10:**
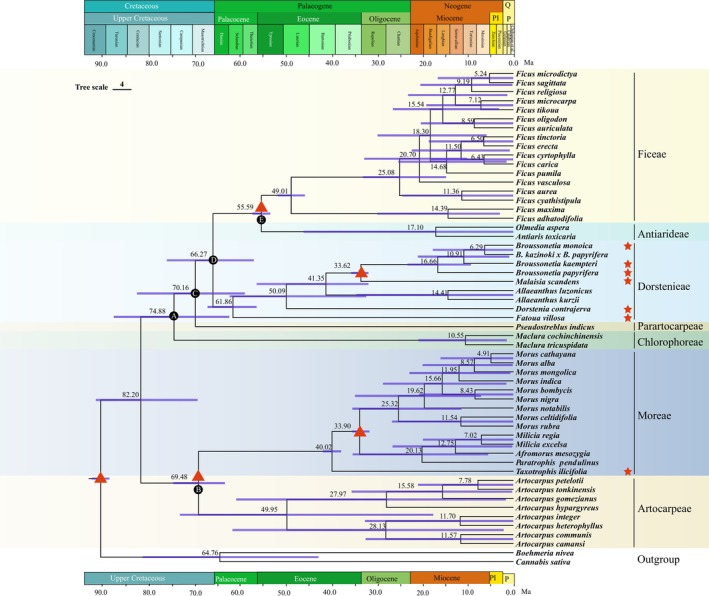
Molecular dating of 53 Moraceae speceis based on plastomes. Red pentagrams mark the seven species sequenced in this study. Red triangles represent calibration points; details are provided in Methods 2.5. Detailed explanations of nodes A, B, C, D, and E are available in Table S7.

According to the BEAST analysis time‐tree (Figure 10), the effective sample sizes (ESS) for all parameters were greater than 200, indicating high reliability of the sampled nodes. The crown node of Moraceae was estimated to have diverged around 82.20 million years ago (Early Cretaceous, 95% HPD = 69.67–92.10 million years ago). Subsequently, Moraceae underwent evolutionary diversifications, forming distinct tribes. Specifically, the Chlorophoreae diverged around 74.88 million years ago (Early Cretaceous, 95% HPD = 62.69–88.15 million years ago), and the Parartocarpeae around 70.16 million years ago (Early Cretaceous, 95% HPD = 59.24–83.03 million years ago). Further divergence led to the emergence of the Dorstenieae around 66.27 million years ago (Paleocene, 95% HPD = 57.27–76.47 million years ago), and the Ficeae and Antiarideae around 55.59 million years ago (Eocene, 95% HPD = 53.64–57.56 million years ago). Concurrently, around 69.48 million years ago (Early Cretaceous, 95% HPD = 63.65–75.04 million years ago), the Artocarpeae and Moreae diverged. Moraceae underwent a series of evolutionary divergences during the late Cretaceous period, forming distinct tribes at various points in time, with detailed node information available in Table S9.

### 3.5 Codon Usage Bias Analysis

In the Moraceae plastome, 52 protein‐coding genes were selected for codon usage bias analysis based on the phylogenetic results and the evolutionary branches Clade A and Clade B, as well as the seven tribes of Moraceae. The selected genes were concatenated, and codon usage bias was calculated for the 53 Moraceae plants. These plants encode 20 amino acids with a total of 64 codons, of which 61 are encoding codons and three are stop codons (UAA, UGA, and UAG) that do not participate in gene encoding. Leucine (Leu) showed the highest frequency of amino acid usage, accounting for 10.67% of all amino acids with 120,862 occurrences; the codon UUA was the most frequently used for leucine, representing 3.55% of usage, while the codon CUC remained one of the least frequently used, at 0.62%. Cysteine (Cys) was the least frequently used amino acid, constituting only 1.12% of total amino acids with 12,718 occurrences. Among these codons, 30 had Relative Synonymous Codon Usage (RSCU) values greater than 1, indicating higher usage frequency, while 34 had RSCU values of 1 or less, indicating lower usage. Notably, of the 30 codons with RSCU values greater than 1, 13 ended with A and 13 with U, and only one ended with G, suggesting a preference for codons ending in A and U (Figure S10 and Table S10).

To explore codon usage patterns among Moraceae chloroplast genomes, we classified the sampled taxa into seven phylogenetic tribes: Moreae, Antiarideae, Dorstenieae, Paratocarpeae, Ficeae, Chlorophorea, and Artocarpeae. RSCU values were calculated and compared across these groups (Figure S11). Our results revealed a generally conserved codon usage pattern across the tribes, with certain codons showing strong preferences. For example, codons such as GCU (Ala), AGA (Arg), ACU (Thr), and AAU (Asn) consistently displayed high RSCU values (> 1.5), indicating preferential usage in the chloroplast genomes of Moraceae. Conversely, codons such as CGC, GCG, UCG, and AGC were underrepresented, with RSCU values typically below 0.6. While most codons showed consistent trends across tribes, several codons exhibited subtle yet notable variation. For instance, UUA (Leu) and AGA (Arg) showed slightly elevated usage in Dorstenieae, while CCC (Pro) and GCG (Ala) were relatively more frequent in Chlorophorea. These findings suggest lineage‐specific codon preference patterns.

Codon usage bias parameters, including T3s, C3s, A3s, G3s, CAI, CBI, Fop, ENC, and GC3s, were calculated for the protein‐coding genes of 53 Moraceae species using the CodonW program, and differences among these species were compared. Overall, there were some variations in codon usage biases across the different species within the Moraceae, but the differences were minor and not statistically significant in terms of codon usage within the plasomes. The analysis revealed the composition of the third base of codons encoding T, C, A, and G as T3s, C3s, A3s, and G3s, respectively. It was found that the content of A/T at the third position of the codons was significantly higher than that of G/C. For instance, the highest T3s content was in *F. villosa* (0.4807), while the lowest was in *M. cochinchinensis* (0.4766). The highest C3s content was observed in *M. excelsa* (0.1637), with the lowest in *D. contrajerva* (0.1583). *D. contrajerva* also had the highest A3s content (0.4446), and *M. excelsa* the lowest (0.4389). The species with the highest G3s content was *M. tricuspidata* (0.1739), and the lowest was *F. villosa* (0.1685). GC3s, indicating the GC content at the third position of codons, showed no significant variation among species. *Milicia excelsa* had the highest GC3s, while *F. villosa* had the lowest, with its content below 25%, averaging around 25.31%.

**FIGURE 11:**
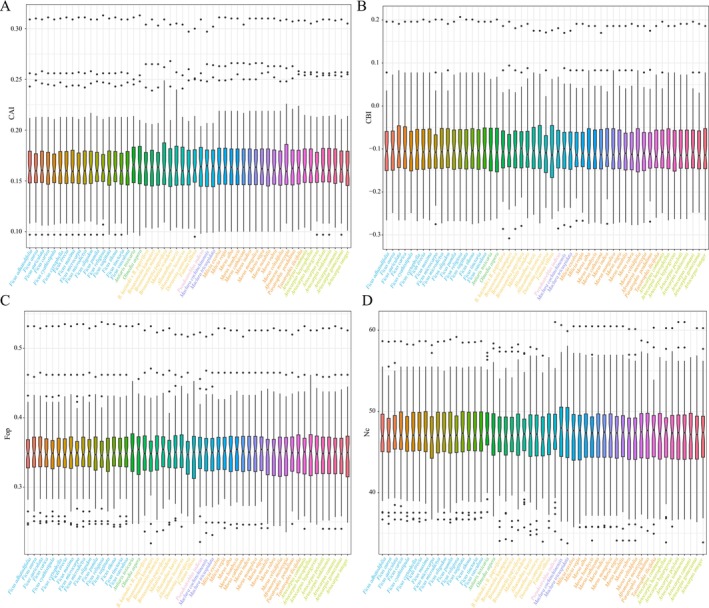
Codon usage statistics of 53 Moraceae plastomes. (A) Codon Adaptation Index (CAI) displays the codon adaptation index statistics, reflecting the relative efficacy of codon usage in terms of translational efficiency. (B) Codon bias index (CBI) shows the codon bias index statistics, which indicate the extent to which codons are preferred or avoided. (C) Frequency of optimal codons (Fop) illustrates the statistics for the frequency of optimal codons, highlighting the most frequently used codons that might enhance translation efficiency. (D) Effective number of codons (ENC) provides statistics on the effective number of codons, which measures codon usage diversity within the genes.

The Codon Adaptation Index (CAI) measures the relative adaptiveness of codons compared with the most optimal codon usage within a gene. The CAI values range from 0 to 1, with higher values indicating stronger adaptiveness to the optimal codons. The CAI values for the 53 Moraceae species studied ranged between 0.165 and 0.168 (Figure 11A). The codon bias index (CBI) assesses the extent to which a subset of optimal codons is used within a gene, with values ranging from −1 to 1; higher CBI values signify a stronger preference for certain codons. The CBI range among the 53 Moraceae species was from −0.113 to −0.104, with the highest in *M. scandens* and the lowest in *D. contrajerva* (Figure 11B). The Frequency of Optimal Codons (Fop) denotes the proportion of optimal codons used among all codons within a gene, with values between 0 and 1, where 1 signifies exclusive use of optimal codons. The Fop values for the 53 Moraceae species ranged from 0.346 to 0.351 (Figure 11C). The effective number of codons (ENC) indicates the diversity of codons used in a gene, ranging from 20 to 61; lower ENC values imply a stronger preference for specific codons. In the 53 Moraceae species, ENC values ranged from 48.24 to 48.87, showing minor variation among species (Figure 11D).

By comparing the codon usage indices of 2756 coding genes extracted from 53 Moraceae species, variations were observed in the values of codon adaptation index (CAI), codon bias index (CBI), and frequency of optimal codons (Fop). Specifically, CAI values ranged from 0.095 to 0.313, with the highest noted in the *psbA* of *M. excelsa* and *F. religiosa* , and the lowest in the *rps18* of *F. villosa* . CBI values varied from −0.308 to 0.207, with the highest observed in *F. religiosa* 's *psbA* and the lowest in the *ccsA* of *B. kaempferi*. Similarly, Fop values ranged from 0.219 to 0.538, with the highest again in the *psbA* of *F. religiosa* and the lowest in the *ccsA* of *B. kaempferi*.

A linear correlation analysis of codon usage bias indices was conducted for 53 Moraceae species. In this analysis, the correlation coefficient (*r*) ranged between [−1, 1]. Coefficients with (|*r*| < 0.3) indicate a low degree of correlation; (0.3 ≤ |*r*| ≤ 0.5) suggest a low to moderate correlation; (0.5 ≤ |*r*| ≤ 0.8) denote a moderate correlation; and (0.8 ≤ |*r*| ≤ 1) reflect a high correlation. Among the findings, T3s showed a positive correlation with A3s but a negative correlation with other codon bias indices. C3s, G3s, CAI, CBI, Fop, Nc, and GC3s demonstrated negative correlations with both T3s and A3s, whereas positive correlations were observed among other indices. Notably, G3s, Nc, and GC3s exhibited a high degree of linear correlation, indicating a significant interrelationship between them (Figure S12).

**FIGURE 12:**
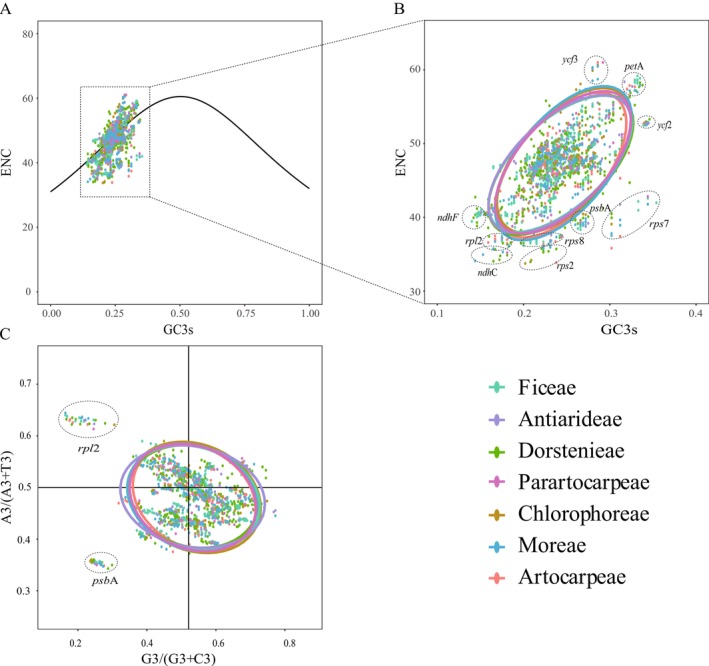
Analysis of ENC‐plot and PR2‐plot for protein‐coding genes in the plastomes of Moraceae species from seven different tribes. (A) ENC‐plot analysis for seven different tribes of the Moraceae species. The expected values are represented by the solid line, and the observed values are indicated by dots. (B) Detailed gene analysis of deviations from the expected values for different tribes of the Moraceae species. (C) PR2‐plot analysis for seven different tribes of the Moraceae species.

**FIGURE 13:**
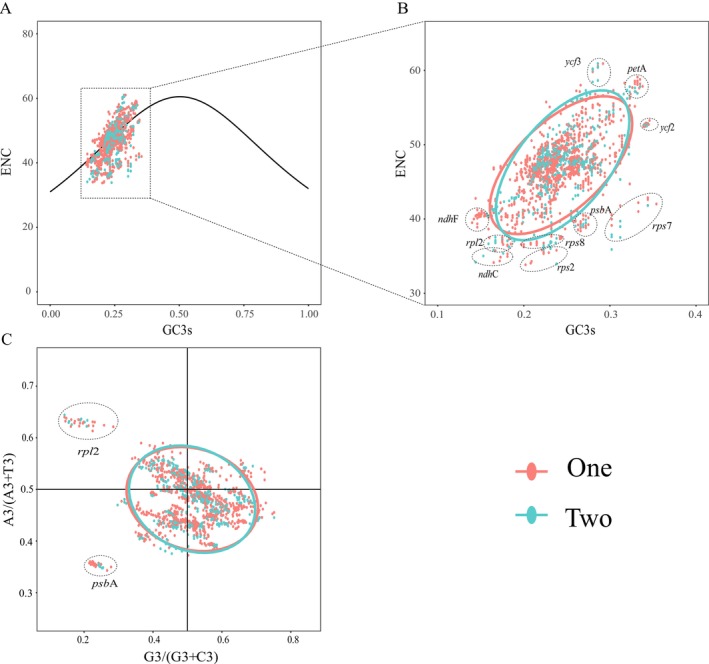
ENC‐plot and PR2‐plot analysis of protein‐coding genes in plastomes from two different clades of the Moraceae. (A) ENC‐plot analysis for two different clades of the Moraceae. The solid line represents the expected values, and dots indicate observed values. (B) Detailed gene analysis of deviations from expected values for two different clades of the Moraceae. (C) PR2‐plot analysis for two different clades of the Moraceae.

Effective number of codons (ENC) values were calculated for 2756 sequences across 52 genes, using ENC = 45 as a threshold to assess codon usage bias. The lowest ENC value observed was 33.75 for the *rps2* in *M. tricuspidata* , indicating a strong codon preference. Conversely, the *ycf3* in *A. tonkinensis* , *P. indicus* , and *A. petelotii* had the highest ENC values at 61, suggesting uniform codon usage across these species. Further analysis by tribal classification within Moraceae revealed that the lowest ENC values were generally for *rps2*, while the highest were for *ycf3*. Exceptions were noted in the Dorstenieae and Ficeae, where the *petA* showed the highest ENC values of 58.25 and 59.17, respectively, and in the Antiarideae, the highest was for the *atpE* at 58.37 and the lowest for the *rpl2* at 37.07. Additionally, in the Moreae and Parartocarpeae, the *ndhC* registered the lowest ENC values at 34.10 and 35.22, respectively. Across phylogenetic clades, *rps2* and *ycf3* maintained their respective lowest and highest ENC trends. Overall, among the Moraceae species' plastomes, ENC values ranged from 33.75 to 61.00, with 2002 sequences having an ENC of 45 or higher and an average ENC of 46.99. ENC‐plots were used to visually represent deviations in synonymous codon usage from randomness, with most genes falling within a narrow range of expected values (Figures 12A and 13A). However, genes such as *rps7*, *rps2*, *psbA*, *rps8*, *ndhC*, *ndhJ*, and *rpl2* showed deviations greater than 0.15, while *clpP*, *ndhI*, and *ycf3* were less than −0.15, indicating a potential impact of natural selection on their codon preferences (Figures 12B and 13B).

The Parity Rule 2 (PR2) plot primarily analyzes codon usage biases at the third position of codons, focusing on the bases A, T, G, and C. It uses the ratio A3s/(A3s + T3s) as the x‐axis and G3s/(G3s + C3s) as the y‐axis to create a scatter plot. Ideally, if a DNA sequence is subject to neutral mutations and selective pressures without bias, genes would adhere to A3s = T3s and G3s = C3s, centering the plot at the intersection of the axes in the PR2‐plot. In an analysis of the Moraceae family, categorized by tribal classification and evolutionary branches, PR2‐plot results indicated sparse gene distribution in the first and second quadrants compared to the more populated third and fourth quadrants, particularly the fourth. This suggested a significant parity bias in the third position of codons among Moraceae genes, with a higher frequency of using T over A and G over C, indicating a preference pattern of T>A and G>C (Figures 12C and 13C).

### 3.6 Analysis of Selective Pressure

**FIGURE 14:**
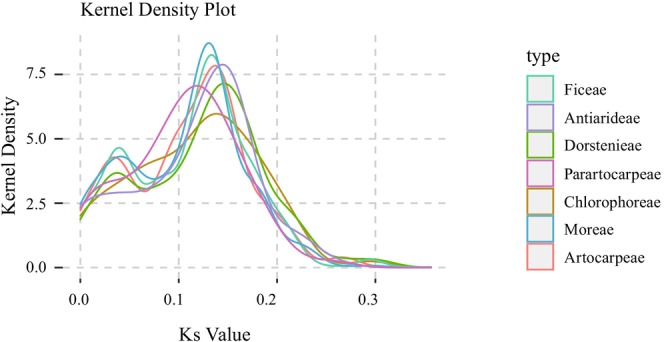
Distribution of *Ks* values for protein‐coding genes shared among the seven different tribes of the Moraceae.

To accurately assess the molecular evolutionary rates across seven tribes of the Moraceae family, this study selected 79 genes common to 40 Moraceae plasomes and calculated synonymous (*Ks*) and nonsynonymous (*Ka*) substitution rates (Figure 14). Comparing the *Ks* values of shared genes across the different tribes and the outgroup, *B. nivea* , revealed variations in overall plastome evolutionary rates. Additionally, genes were categorized based on their function within the plastomes into three groups: self‐replicating genes, photosynthesis‐related genes, and other functional genes (Table S11). The distribution of *Ks* values generally showed dual peaks across most tribes. In contrast, the Chlorophoreae exhibited a lower evolutionary rate compared to others, while the Parartocarpeae's rates tended to shift to the left, suggesting a faster rate (Figure 14).

**FIGURE 15:**
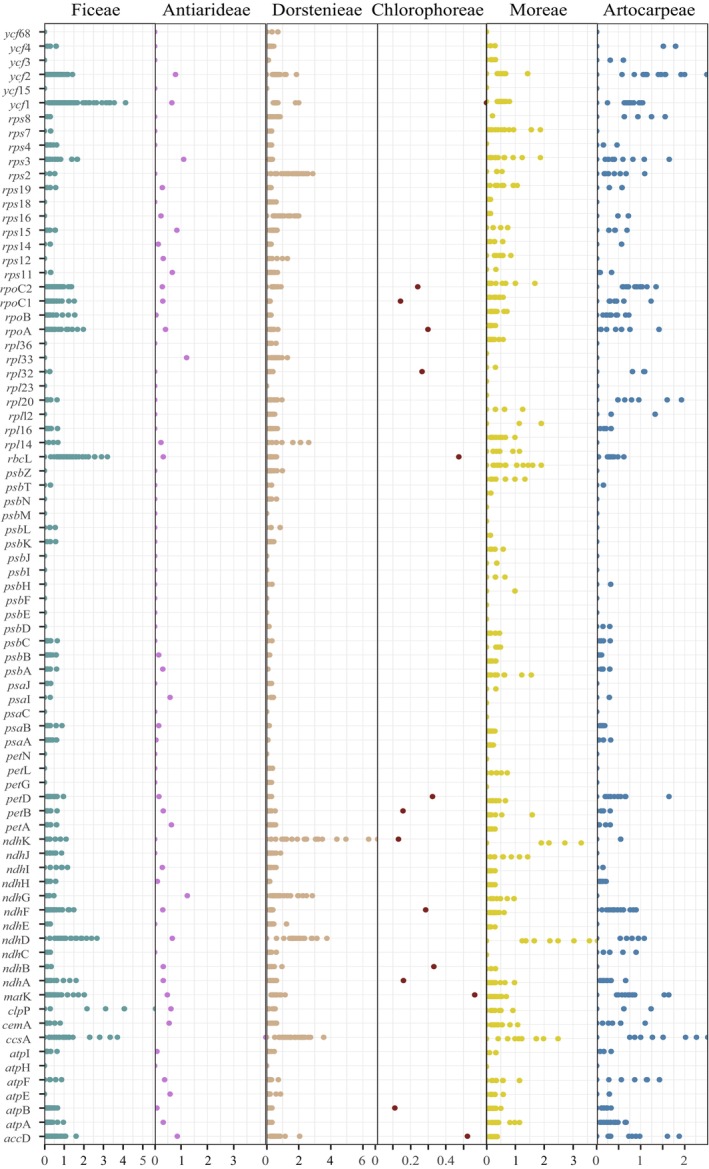
Analysis of selective pressure on genes in the seven tribes of the Moraceae.

**FIGURE 16:**
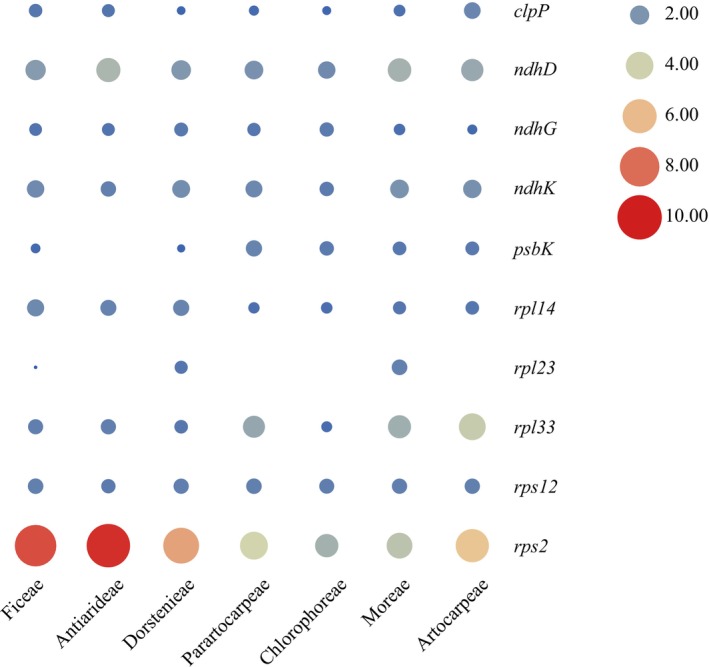
Heatmap of genes with selection pressure greater than 1 within each tribe of Moraceae.

An evolutionary rate analysis of 79 shared protein‐coding genes across 53 Moraceae species revealed substantial differences in evolutionary rates between genes (Figure 15). Most genes in the genome exhibited a *Ka/Ks* ratio less than 1, indicating purifying selection, with notable positive selection observed in the *rps2*. The genes *rps2*, *rps12*, *ndhK*, and *ndhD* demonstrated *Ka/Ks* ratios greater than 1 across various tribes, suggesting positive selection. Additionally, the genes *clpP*, *ndhG*, *psbK*, *rpl14*, and *rpl23* showed *Ka/Ks* ratios greater than 1 in certain tribes but less than 1 in others. Specifically, *clpP* underwent positive selection in the Artocarpeae and Ficeae, *ndhG* in the Chlorophoreae and Parartocarpeae, *psbK* across the Artocarpeae, Chlorophoreae, Moreae, and Parartocarpeae, and *rpl14* in the Artocarpeae, Chlorophoreae, Ficeae, and Antiarideae. The *rpl23* was positively selected in the Moreae and underwent purifying selection in others. All remaining genes experienced purifying selection, with evolutionary rates below 1 (Figure 16). Based on clade division, the *ndhD*, *ndhK*, *rpl14*, *psbK*, *rps12*, and *rps2* displayed *Ka/Ks* ratios above 1 in both Clade A and Clade B, indicating positive selection. The *psbK* and *clpP* also had *Ka/Ks* ratios above 1 in Clade B, while *ndhG* showed a similar ratio in Clade A. Comparisons with the outgroup *B. nivea* revealed no significant relationships either among functionally classified genes within different tribes or among different clades (Figure S13 and Table S12).

## 4 Discussion

### 4.1 Characteristics of the Plastome in the Moraceae

The Moraceae is widely distributed across the globe, primarily in tropical and subtropical regions, with some species extending into temperate zones. Due to their extensive distribution and diversity, the plastome of many species within the Moraceae remains unreported, hindering comprehensive understanding. This study employed second‐generation sequencing technology to perform whole plastome sequencing on seven Moraceae species from the genera *Broussonetia*, *Malaisia*, *Dorstenia*, *Fatoua*, and *Taxotrophis*. The plastome sequences of *Dorstenia contrajerva* , *Fatoua villosa* , and *Taxotrophis ilicifolia* are reported here for the first time, filling gaps in Moraceae plastome research and providing a foundation for future phylogenetic, genetic evolution, and plastome analyses. Our analyses of the plastomes of 53 Moraceae species revealed that the genome structure of the seven newly sequenced species was consistent, featuring a typical quadripartite structure (LSC, IR, SSC) with sizes ranging from 158,459 bp to 162,594 bp and GC content between 35.3% and 36.4%. The IR region had the highest GC content, while the SSC region had the lowest. The single‐copy regions and nonprotein‐coding regions exhibited greater variability. The results indicated that the general characteristics of Moraceae plastomes are similar to those of other land plants (Barrett et al. 2014; Tiller and Bock 2014; Liu et al. 2018; Huang et al. 2023).

The plastomes of Moraceae species are generally conserved in terms of gene content, number, and structure. This study analyzed the plastomes of 53 Moraceae species and found that the numbers of protein‐coding genes, tRNA, and rRNA were relatively stable, with rRNA showing the least variation, consistent with other terrestrial angiosperms. The study revealed that all 53 Moraceae species lacked the *infA* and possessed a nonfunctional *Ψycf1*. Additionally, the *rpl22* was either absent or shortened in some species, and premature stop codons were observed in the *ycf15* of certain species. These phenomena are not unique to Moraceae; similar losses of the *rpl22* gene in Passifloraceae (Shrestha et al. 2020) and Rosaceae (Jansen et al. 2010), as well as the *infA* gene in *Solanum* (Millen et al. 2001), were observed, suggesting that gene variation is common in plastomes of angiosperms. The consistent absence of the *infA* gene across all 49 Moraceae species suggests that this gene, which is completely lost or exists only as a pseudogene in many angiosperms (Gantt et al. 1991; Bausher et al. 2006; Lee et al. 2006), may have been transferred to the nuclear genome. Specifically, the loss of the *rpl22* was observed in species such as *Broussonetia kaempferi* and *Broussonetia monoica*, while a shortened version of the gene was found in *Malaisia scandens* and *Allaeanthus kurzii*. These gene losses or alterations may indicate migration to the nuclear genome (Bausher et al. 2006; Gantt et al. 1991; Lee et al. 2006), reflecting the prevalence of gene transfer and structural variation within plastomes. Furthermore, the *ycf15* in species such as *Milicia excelsa* and *Taxotrophis ilicifolia* was notably shorter, resembling the premature termination observed in the *ycf15* of other angiosperms (Fajardo et al. 2013; Hu et al. 2016), including *Dioscaryon*, *Cardicaryon*, and *Vaccinium macrocarpon* . However, its precise function remains unclear and warrants further investigation (Gao et al. 2017; Li et al. 2021). Moreover, the *ycf1* in the plastomes of the 53 Moraceae species existed in two copies: one complete and one pseudogene. The *Ψycf1* spanned the JSB region and has lost its function, highlighting structural changes in plastomes during evolution and the mechanisms behind gene function loss and pseudogene formation.

### 4.2 DNA Barcoding in Moraceae

This study examined nucleotide polymorphisms and insertion/deletion events in the plastomes of 53 Moraceae species, uncovering the high conservation of the IR region and the variability of the LSC and SSC regions. These findings corroborate previous research, reinforcing the notion that the unique inverted repeat structure of the IR region enables effective repair of variations, thus maintaining stability. Despite the potential of plastid genomes as “super barcodes” for species differentiation (Wu et al. 2021), practical applications still depend on short DNA fragments due to incomplete data and resource constraints. The study identified highly variable regions (*trnC‐petN*, *rps4‐trnT*, *ndhF‐rpl32*, *rps15‐ycf1*) and a highly variable gene (*ycf1*) that present potential DNA barcodes for Moraceae species. Notably, the *ycf1*, owing to its high nucleotide diversity, emerges as a suitable core barcode, consistent with prior studies that highlight the significance of *ycf1* in plant taxonomy (Dong et al. 2012, 2015). Additionally, the hypervariable regions of plastomes provide selectable DNA barcodes (Hebert et al. 2003; Chase et al. 2005; Schindel and Miller 2005; Lahaye et al. 2008; Seberg and Petersen 2009), enhancing the accuracy of species identification and promoting research on plant evolutionary relationships (Figures S2–S7). Moreover, the identification of 5350 dispersed and tandem repeat sequences and 4751 simple sequence repeats offers tremendous information for screening of molecular markers in Moraceae, as the utility of repeat sequences in genetic research has been emphasized by many studies. In summary, this study elucidated the variability characteristics of Moraceae plastomes, providing novel tools for taxonomy and molecular marker research while validating the outcomes of previous studies through comparative analysis.

### 4.3 Exploring the Phylogenetic Relationships and Evolutionary History of Moraceae

The evolution of Moraceae, as revealed through phylogenetic analysis, demonstrated a complex and diverse evolutionary relationship. Our study analyzed plastome data from 53 Moraceae species, discovering that despite variations in phylogenetic signals among different datasets, all results consistently supported the monophyly of the seven tribes of Moraceae, which were divided into two major clades: Clade A and Clade B. Clade A comprised Dorstenieae, Ficeae, Antiarideae, Parartocarpeae, and Chlorophoreae, while Clade B included Artocarpeae and Moreae. This study was the first to report the plastome genomes of *Dorstenia contrajerva* and *Fatoua villosa* , revealing their placement in different genera within the tribe Dorstenieae, forming a monophyletic sister branch that aligns with the systematic classification of Moraceae. Regarding the relationship between *Artocarpus hypargyreus* and *Artocarpus gomezianus*, although differences existed in sequence matrices among various datasets, the phylogenetic tree constructed from the full‐length matrix exhibited higher reliability, supporting a close relationship between *Artocarpus gomezianus* and *Artocarpus tonkinensis* (Lin et al. 2021). Additionally, *Broussonetia monoica* and the hybrid *B. kazinoki × B. papyrifera* formed sister species, corroborating the hypothesis that *Broussonetia monoica* results from hybridization between dioecious diploids and polyploids (Kuo et al. 2022). Moreover, the monophyly of *Malaisia scandens*, *Allaeanthus kurzii*, and *Allaeanthus luzonicus*, along with the sister species relationship between *Olmedia aspera* and *Antiaris toxicaria*, further validated previous research conclusions. However, within Ficeae, certain nodes displayed low support values, particularly for species such as *F. carica* , *F. cyrtophylla*, *F. erecta* , *F. macrocarpa* , *F. religiosa* , *F. sagittata*, *F. tikoua*, and *F. tinctoria* . These nodes, despite being part of the same tribe, showed weak support due to several potential biological, evolutionary, and technical factors. One possible reason could be the lack of resolution in plastid data, which may not contain enough variation in certain regions to clearly resolve relationships among these species (Davis et al. 2014). Another contributing factor could be rapid radiation within Ficeae, where the diversification of species occurred too quickly for the plastome data to capture clear phylogenetic signals (Cauz‐Santos et al. 2025). Additionally, the limitations of plastome data in capturing the full extent of genetic variation within this tribe might have contributed to the observed low support (Maddison 1997). Further research incorporating additional markers or more comprehensive sequencing techniques may help clarify these relationships. As a result, these challenges highlight the need for further research incorporating additional markers or more comprehensive sequencing techniques, which may help clarify these relationships. In contrast, the conservation of plastome genomes provides robust support for the phylogenetic relationships among the tribes of Moraceae. This study, based on plastome data and multiple phylogenetic methods, offers a more robust understanding of the evolutionary relationships within Moraceae, deepening our comprehension of the evolutionary processes of these plants. The historical evolution of the Moraceae indicated that their crown group formation occurred approximately 82.20 million years ago, slightly earlier than the estimates by Zhang (2019) and Zetega et al. (2005). The crown group nodes for the Artocarpeae and Ficeae tribes were established around 49.95 million years and 49.01 million years ago, respectively, which are younger and more accurate than previous estimates. Similarly, the crown group nodes for the Dorstenieae and Moreae tribes were precisely dated to 61.86 million years and 40.02 million years ago, respectively (Zerega et al. 2005; Zhang 2019). Discrepancies among different studies may arise from variations in datasets, analytical methods, or models employed. Nevertheless, the divergence times reported in our study are generally consistent with previous findings while providing more precise confidence intervals. The Moraceae underwent significant evolution and expansion from the early Cretaceous to the Eocene, particularly during the Eocene epoch, when multiple tribes diversified, indicating a trend of species diversification. This increase in plant diversity during this period might be closely related to the extinction of herbivorous animals, changes in soil nutrients, and climate warming (Jaramillo et al. 2006, 2010; Jud et al. 2018; Carvalho et al. 2021).

### 4.4 Factors Influencing Codon Usage Bias in Moraceae

Throughout their evolution, vascular plants have developed specific preferences for certain codons. This study analyzed 53 Moraceae species and identified a significant bias in codon usage. The analysis showed that leucine had the highest coding frequency, while cysteine demonstrated the lowest. Codons ending in A and U were used more frequently, consistent with the common codon termination patterns observed in plant organellar genomes (Campbell and Gowri 1990). By comparing the RSCU values across seven representative tribes of Moraceae, we observed that while the overall codon usage patterns are relatively consistent, certain codons show variation among tribes. This reflects both the conserved and divergent aspects of codon usage in the chloroplast genomes of Moraceae. From a phylogenetic perspective, the conservation and divergence in codon preferences may serve as auxiliary indicators of evolutionary relationships among tribes. For instance, Ficeae and Dorstenieae exhibit highly similar RSCU values for most codons, which may reflect a closer phylogenetic relationship. Such evolutionary stability in codon usage patterns has also been confirmed in other studies (Wang et al. 2022). Additionally, 13 optimal codons were identified: UUG, CUU, UCA, CCA, ACU, ACA, GCU, UAA, CAA, AAU, GAU, CGA, and GGA. These optimal codons also tend to end in A and U. Codon Usage Bias (CUB) also plays a critical role in plastome research. CUB refers to the nonrandom patterns of synonymous codon usage when encoding proteins (Grantham et al. 1980; Toshimichi 1985; Salim and Cavalcanti 2008). In plastomes, codon usage bias is influenced by various factors, including gene expression levels, mutational pressure, and natural selection (Bulmer 1991; Drummond et al. 2006; Plotkin and Kudla 2011; Camiolo et al. 2015). By studying codon usage bias in plastomes, researchers can uncover the selective pressures experienced by plastome genes during different evolutionary processes, further enhancing the understanding of plastome gene expression and function. The bias in codon usage is primarily influenced by factors such as mutation and natural selection (Ruth and Dmitri 2008; Chakraborty et al. 2020; Parvathy et al. 2022). Mutations, as a key driving force in genome evolution, significantly affect the development of codon bias. Natural selection, on the other hand, optimizes the frequency of certain codons based on their advantages for gene expression and function. ENC‐plot analysis revealed that the expected and observed values for most genes in the Moraceae family were closely aligned, indicating that mutation has a significant impact on codon bias. However, for a few genes, such as *rps7*, *rps2*, and *psbA*, the observed values differ significantly from the expected values, suggesting a stronger influence of natural selection. Additionally, PR2‐plot analysis supports the conclusion that mutation plays a major role, with natural selection acting as a secondary factor. Overall, the formation of codon bias in Moraceae is primarily driven by the combined effects of mutation and natural selection, with mutation playing a dominant role.

Additionally, the *ycf3* gene in both branches of Moraceae has an ENC value of 61, indicating a complete lack of codon usage bias. This absence of codon preference is often linked to relatively low gene expression, since highly expressed genes usually experience translational selection favoring optimal codons to improve efficiency and accuracy. Although direct transcriptome data for *ycf3* in Moraceae species is currently unavailable, studies in tobacco ( *Nicotiana tabacum* , Solanaceae) show that *ycf3* has low normalized expression (signal intensity around 0.5) in leaf transcriptomes, suggesting that chloroplast‐encoded *ycf3* tends to be expressed at low levels even in representative angiosperms (Vranová et al. 2002). This pattern supports the idea that the lack of codon bias in *ycf3* may result from its low expression, leading to little selective pressure for translational optimization. Moreover, the gene's *Ka/Ks* ratio of 0.1036 indicates strong purifying selection at the protein level, suggesting that the gene's function is highly conserved. This means that the lack of codon bias in *ycf3* is not due to relaxed selective constraints at synonymous sites, but more likely due to low expression levels causing weak translational selection. Taken together, these findings suggest that the absence of codon usage bias in *ycf3* arises from a combination of low expression and weak selection for translational efficiency, despite strong conservation at the protein level. This phenomenon is also found in species of *Diospyros* (Huang et al. 2023). The *ycf3* gene is associated with the thylakoid membrane and contains multiple tetratricopeptide repeat (TPR) domains, which mediate protein–protein interactions (Naver et al. 2001; Nellaepalli et al. 2018). The complex function of the *ycf3* gene may require diverse codons to encode different amino acids, contributing to the absence of significant codon bias.

### 4.5 Differential Evolutionary Rates in Moraceae

In this study, we found that the evolutionary rates among different tribes were highly similar, but there were significant differences between individual genes. Most protein‐coding genes had *Ka/Ks* values less than 1, indicating purifying selection. However, the *rps2*, *rps12*, *ndhK*, and *ndhD* exhibited evolutionary rates greater than 1 across all tribes, with *rps2* showing the most significant positive selection. By analyzing the ratio of nonsynonymous to synonymous substitutions (*Ka/Ks*), researchers can explore the driving forces and mechanisms underlying biological evolution (Nei 2005; Schierup et al. 2008; Jeffares et al. 2015). Compared with nuclear genomes, plastomes have a lower rate of synonymous substitutions (Wolfe et al. 1987; Gaut 1998; Drouin et al. 2008); however, the evolutionary rates vary significantly across different species and gene regions. These rates are influenced by multiple factors, including mutations, selective pressure, and genetic drift. Analyzing the evolutionary rates of plastomes is crucial for a comprehensive understanding of plant evolutionary processes.

The *rps* gene family, involved in the assembly and function of chloroplast ribosomes, includes members such as *rps19* and *rps8* from the Ampelopsideae tribe, both of which have also undergone strong positive selection (Luo 2023). Specifically, both *rps2* and *rps12* exhibit positive selection in Moraceae. The positive selection of *rps* genes could be linked to their critical role in maintaining efficient chloroplast function and enhancing photosynthetic capacity, particularly under environmental stresses such as light intensity or temperature fluctuations (Saha et al. 2017). Moraceae spp. are widely distributed in tropical and subtropical regions (e.g., the Artocarpeae spp. mainly occur in the Neotropical and Paleotropical regions [Zerega et al. 2010], and the Antiarieae spp. are distributed in tropical regions of the Americas). They are exposed to high‐temperature and intense‐light environments. High temperatures and intense light tend to cause protein misfolding (Chen et al. 2022). The positive selection of *rps* genes may optimize ribosome structure to improve translation efficiency, ensuring the rapid synthesis of photosynthesis‐related proteins. This adaptation enables plants to better cope with varying conditions like high light or drought. The *ndh* gene family, participating in Photosystem I cyclic electron transport and chlororespiration (Whelan et al. 1992; Peng et al. 2011), shows numerous synonymous mutation sites in genes like *ndhF* in *Lagerstroemia* (Wang et al. 2023) and *ndhA* in Ampelopsideae (Luo 2023). Although all tribes of Moraceae are generally distributed in warm and humid tropical to subtropical regions, there are differences in the microhabitats among different tribes. The positive selection of *ndh* genes may help Moraceae plants maintain energy metabolism balance in fluctuating environments such as drought and intense light by optimizing the efficiency of the electron transport chain or enhancing chloroplast respiration (Burrows et al. 1998; Sabater 2021).

From a biogeographic dispersal standpoint, the migration trajectories and distributional patterns of Moraceae species provide compelling evidence for the adaptive significance of the positively selected genes discussed above. A case in point is *Artocarpus*: during its range expansion from Borneo to Asia–Oceania (Zerega et al. 2010), the genus undoubtedly underwent environmental filtering mediated by regional microhabitat variations—including precipitation regimes, edaphic properties, and associated biotic communities. Such transcontinental dispersal events subjected these plants to novel combinations of environmental stressors in colonized habitats. Crucially, this dispersal‐induced environmental heterogeneity likely intensified selective pressures driving functional refinement of *rps* and *ndh* genes. These findings suggest that the positive selection of *rps2*, *rps12*, *ndhK*, and *ndhD* could be associated with adaptive responses to environmental stresses (Ruhlman et al. 2015; Saha et al. 2017). These genes are integral to critical processes like photosynthesis, energy regulation, and oxidative stress management (Ruhlman et al. 2015).

The plastomes of various tribes in Moraceae exhibit a pattern of “core conservation with local adaptation.” Compared with the outgroup *Boehmeria nivea* , there is no significant difference in gene function classification and evolutionary rate among different tribes, reflecting a relatively conservative evolutionary pattern in Moraceae plants during long‐term evolution. Although tribes of Moraceae have a wide geographical distribution (from tropical America to temperate Asia), most occupy similar mid‐to‐high vegetation niches (trees or shrubs). Similar demands for light and water have led to convergent purifying selection pressure on core genes. In contrast, the positive selection of *rps* and *ndh* genes belongs to “fine‐tuning adaptation,” which is a molecular strategy for each tribe to cope with microenvironmental differences (such as drought cycles and light intensity) under similar macrohabitats (Drouin et al. 2008).

## 5 Conclusion

This study advances the understanding of Moraceae phylogenomics by sequencing seven complete plastomes and analyzing data from an additional 46 plastomes at the tribal level. Our findings confirm the monophyly of the seven tribes within Moraceae and establish a robust phylogenetic framework with time divergence dated approximately 82.20 million years ago. We identified 10 highly variable regions, along with thousands of repeats and SSRs, providing valuable molecular markers for species identification and phylogenetic studies. The evolutionary analysis revealed positive selection in key genes and a codon usage bias influenced by both mutation and selection. Future research should focus on the adaptive mechanisms and genetic underpinnings that enable Moraceae species to thrive in diverse environments. Our genomic resources will support genetic engineering and germplasm exploration, thereby enhancing the utility of Moraceae species in food, medicine, ecological restoration, and industrial applications. This study laid a solid foundation for further phylogeographic and population genetic research within this versatile plant family.

## Author Contributions

**Li‐Na Zhou:** data curation (lead), formal analysis (lead), investigation (lead), methodology (lead), software (lead). **Shi‐Zhen Wu:** data curation (equal), formal analysis (equal), methodology (equal), software (equal), writing – review and editing (equal). **Qing Ma:** writing – review and editing (equal). **Xiao‐Wen Jia:** data curation (equal), formal analysis (equal), software (equal), supervision (equal). **Pan Li:** data curation (equal), investigation (lead), funding acquisition (lead), resources (equal), writing – original draft (equal), writing – review and editing (equal). **Xin‐Jie Jin:** conceptualization (lead), writing – original draft (lead), writing – review and editing (lead). **Yong‐Hua Zhang:** conceptualization (lead), funding acquisition (lead), investigation (lead), project administration (lead), resources (lead), validation (lead), writing – review and editing (lead).

## Conflicts of Interest

The authors declare no conflicts of interest.

## Supporting information


**Table S1:** Basic information of Moraceae accessions used for phylogenetic analyses.

**Table S2:** Features of plastomes of 53 Moraceae species.

**Table S3:** List of genes in the plastomes of Moraceae.

**Table S4:** Comparison of GC content of each part.

**Table S5:** (A) Analysis of the overall sliding window of 49 Moraceae plastomes. (B) Analysis of nucleotide polymorphism of protein coding gene.

**Table S6:** Number and types of repeat sequences in 37 Moraceae cp genomes and the distribution of simple sequence repeats (SSR), dispersed repeat sequence (DRS), and tandem repeat sequence (TRS) in 53 Moraceae cp genomes.

**Table S7:** (A) Summary of SSR raw data (these yellow ones are mono—and poly—dinucleotide repeats). (B) Summary of Scattered in repeating sequences raw data. (C) Summary of tandem repeats raw data.

**Table S8:** Characteristics of the three matrices.

**Table S9:** Summary of key node results for divergence time estimation based under a Bayesian approach implemented in BEAST for Moraceae.

**Table S10:** (A) The condon type, number of codons, amino acid, and the relative synonymous codon usage (RSCU). (B) Analysis of the difference in preference coefficients within the genus Moraceae. (C) ENC, PR2 plots and Codon preference factor datas of protein‐coding genes in cp genomes of 53 Moraceae species. (D) Screening of 53 optimal codons from Moraceae plants.

**Table S11:** Common genes in the plastid genomes of 53 Moraceae plants.

**Table S12:** (A) Species‐level pairwise coding sequence Ka/Ks analysis data. (B) The Ka/Ks values of CDS genes among different tribes and clades of Moraceae. (C) The Ka/Ks values of three genes with different functions among different tribes and different clades of Moraceae. (D) The Ka/Ks values in different tribes and clades of Moraceae among the three types of genes with different functions.

**Appendix S1:** Supporting Information.

## Data Availability

The plastomes of seven Moraceae species from the genera *Broussonetia*, *Malaisia*, *Dorstenia*, *Fatoua*, and *Taxotrophis* generated in this study are available in the NCBI GenBank repository (details in Table S1). Specifically, for *Broussonetia*, three species with GenBank accession numbers are PP584596, PP584592, and PP584595, respectively. For *Malaisia scandens*, the GenBank accession number is PP584597. For *Dorstenia contrajerva* , the GenBank accession number is PP584593. For *Fatoua villosa* , the GenBank accession number is PP584594. For *Taxotrophis ilicifolia*, the GenBank accession number is PP584598. Data and code supporting the conclusions of this study are available in the NCBI GenBank repository, and the supplementary data can be found in Table S1.
