# Supplementary material for: Phylogenomic Analyses and DNA Barcoding Development Within Moraceae: Insights Into Genomic Features, Mutational Hotspots, and Adaptive Evolution
Source: Ecol Evol. 2025 Nov 9;15(11):e72399. doi: 10.1002/ece3.72399 (PMC12597254; doi:10.1002/ece3.72399)
Supplement: Supplementary file 13 — Appendix S1: Supporting Information. [file ECE3-15-e72399-s007.docx]

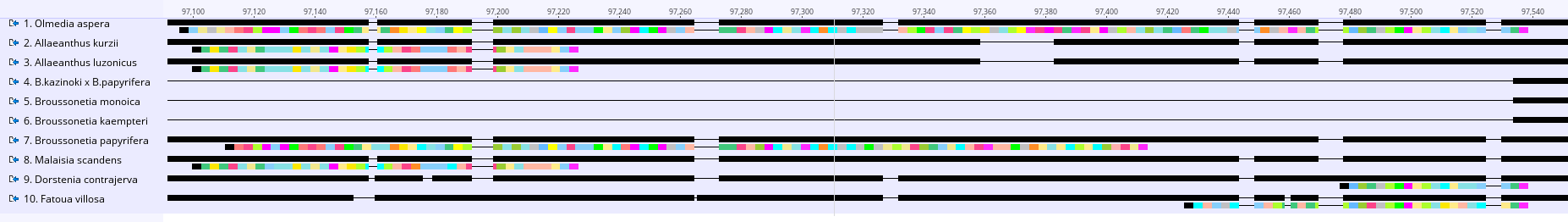


**Figure S1** *rpl*22 Gene deletion and variation in nine species of Dorstenieae plants．


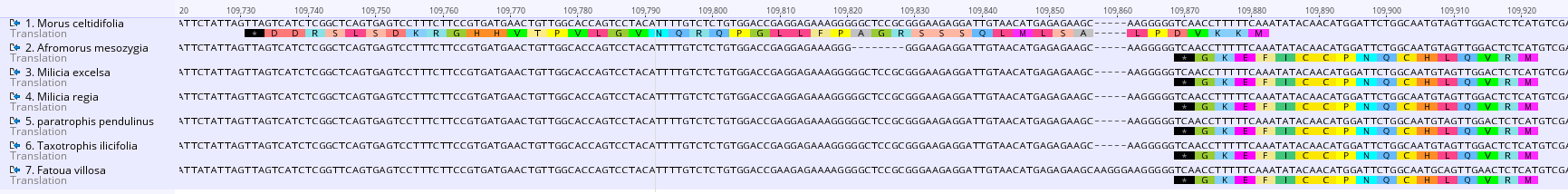


**Figure S2** Premature stop codon occurrence in the *ycf*15 gene．


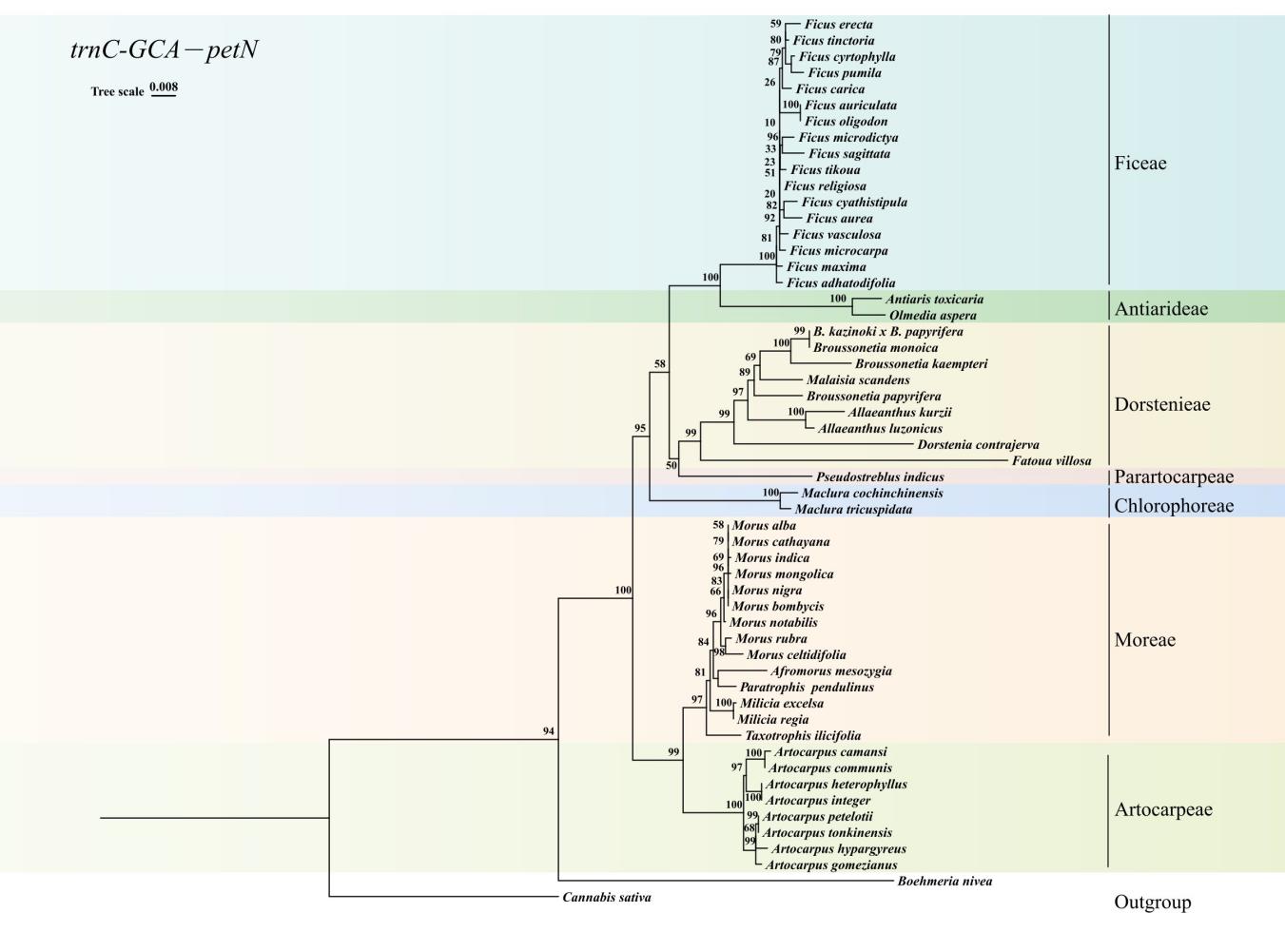


**Figure S3** ML phylogenetic tree constructed based on the *trnC*-GCA－*petN* region


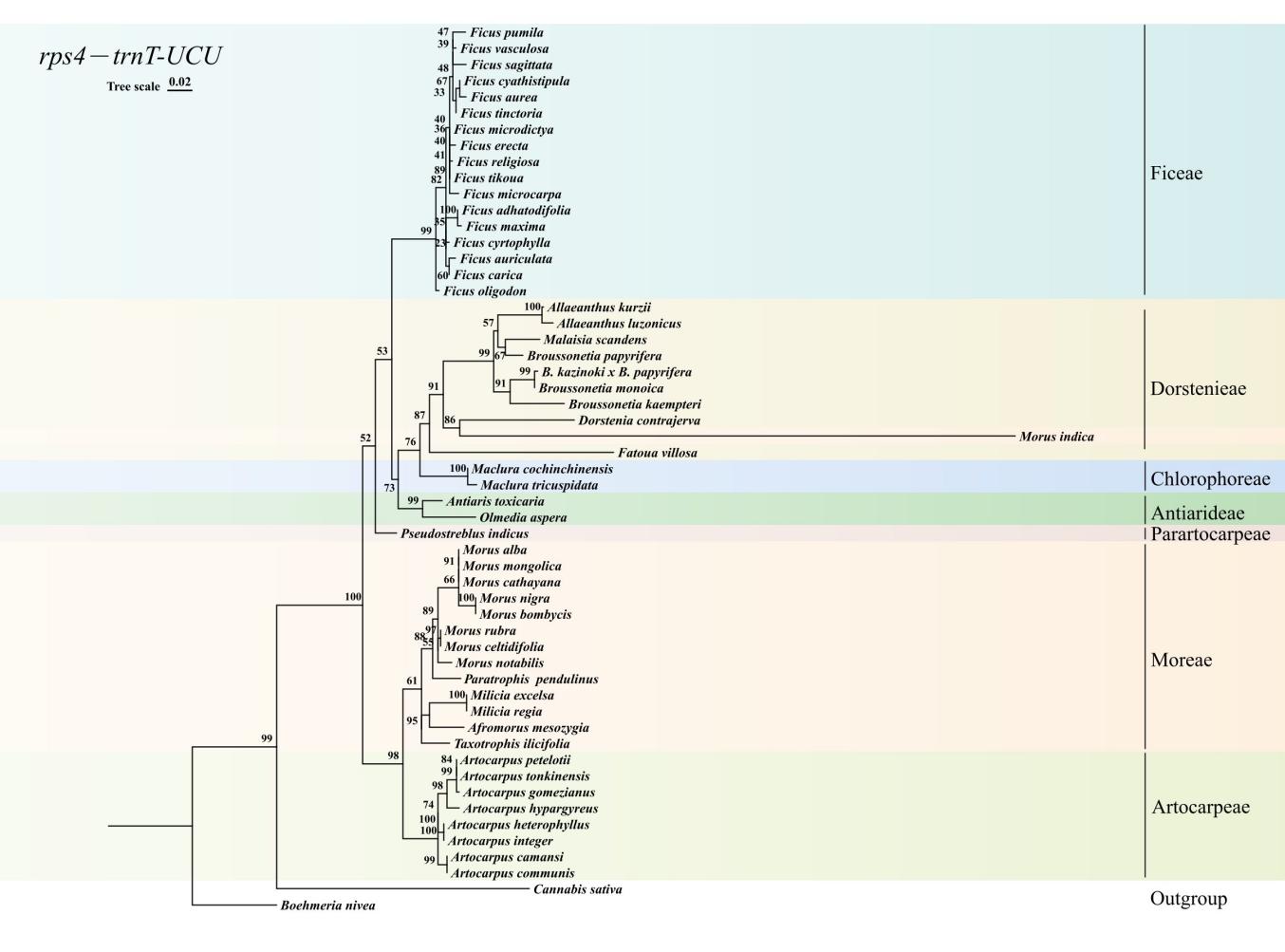


**Figure S4** ML phylogenetic tree constructed based on the *rps4－trnT-*UCU region


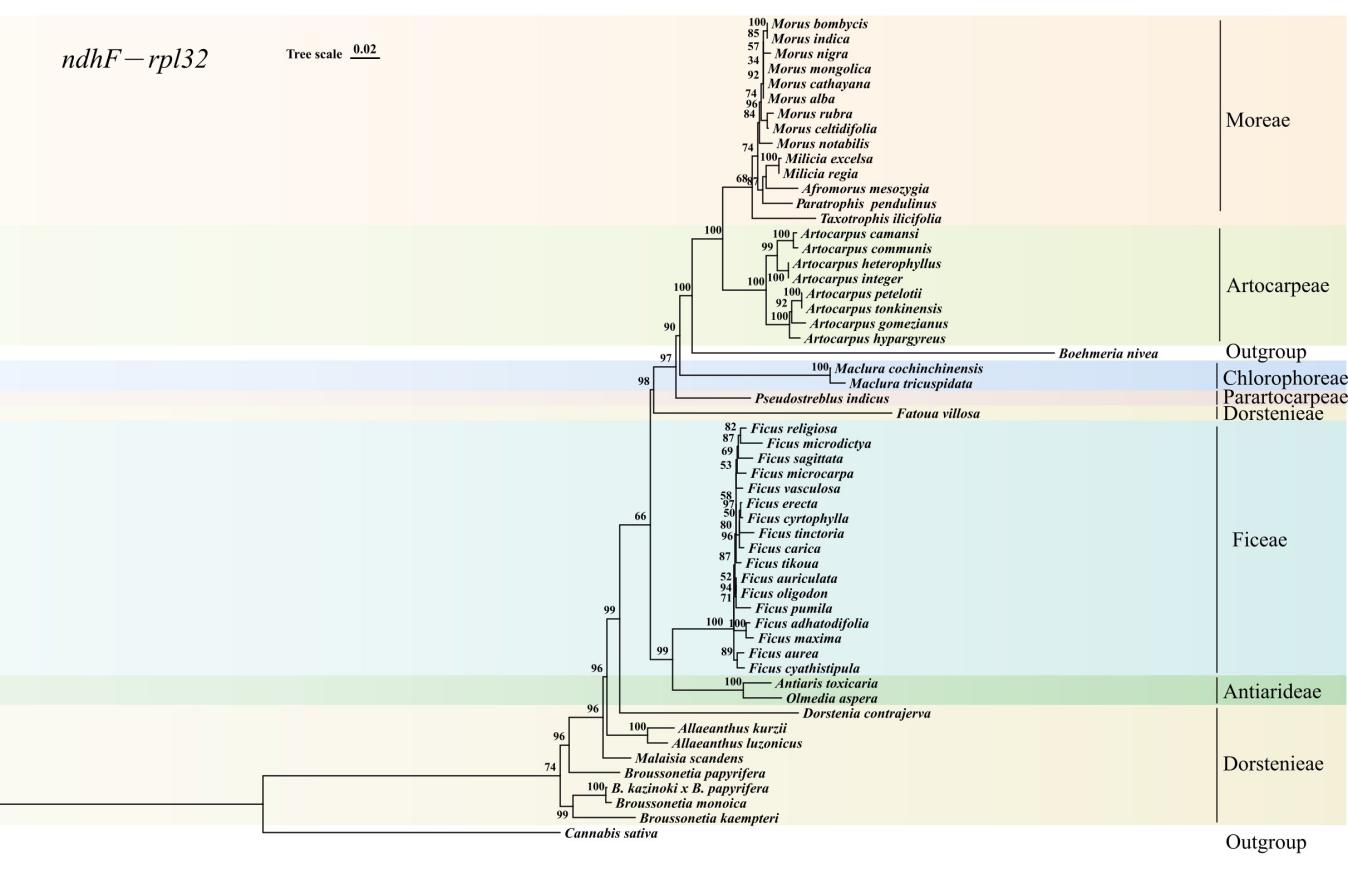


**Figure S5** ML phylogenetic tree constructed based on the *ndhF-rpl32* region


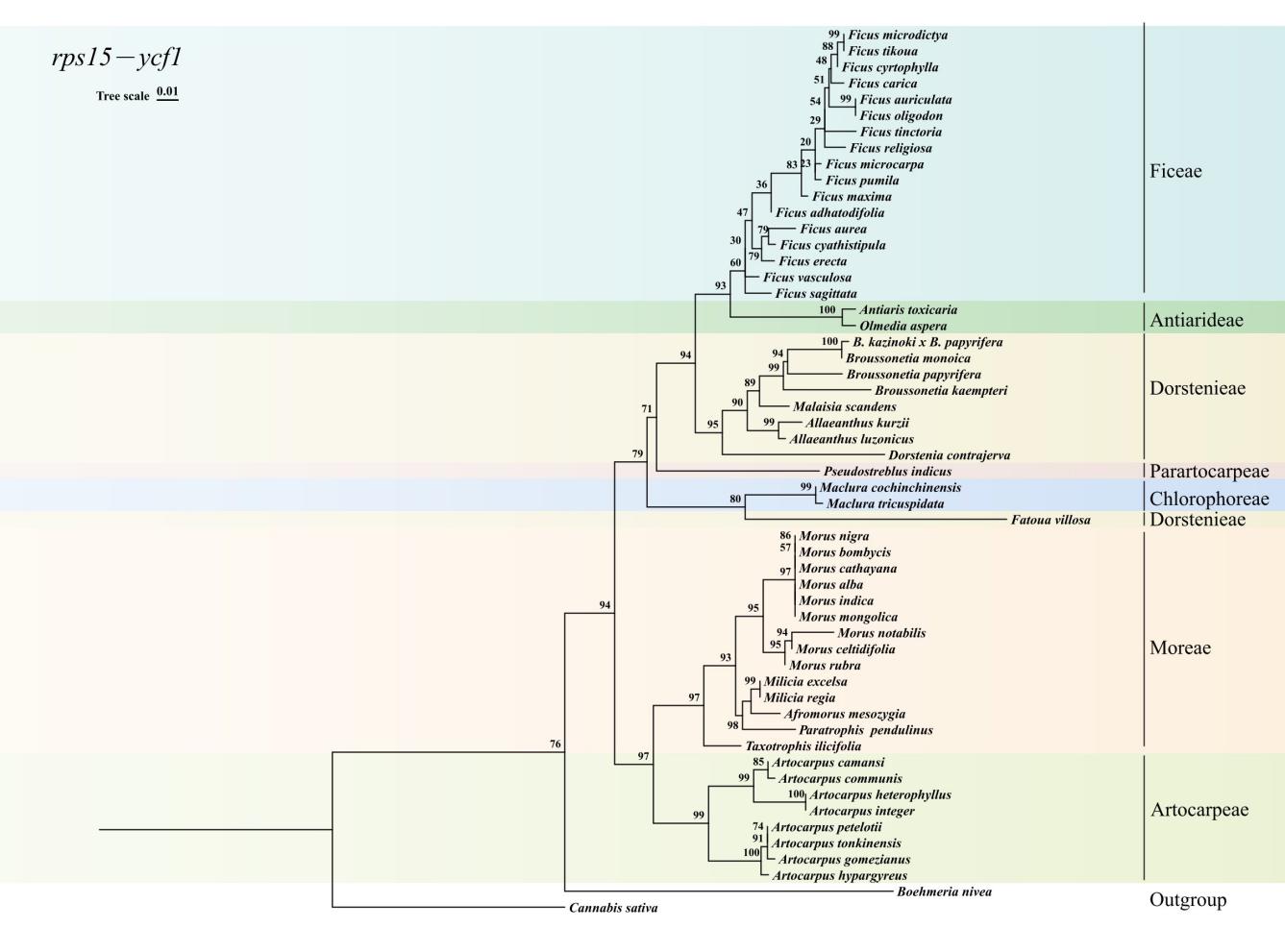


**Figure S6** ML phylogenetic tree constructed based on the *rps15－ycf1* region


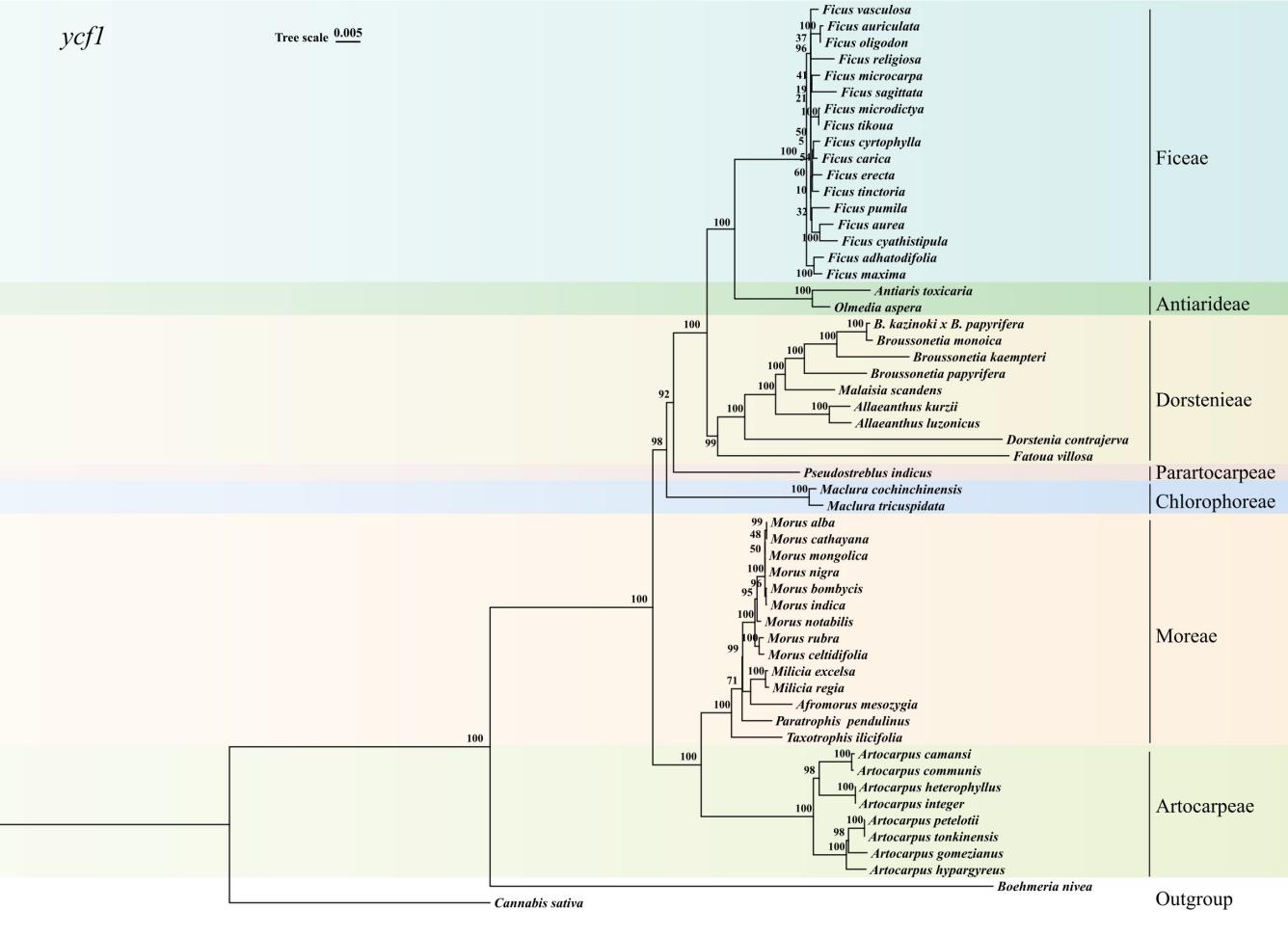


**Figure S7** ML phylogenetic tree constructed based on the *ycf1* region


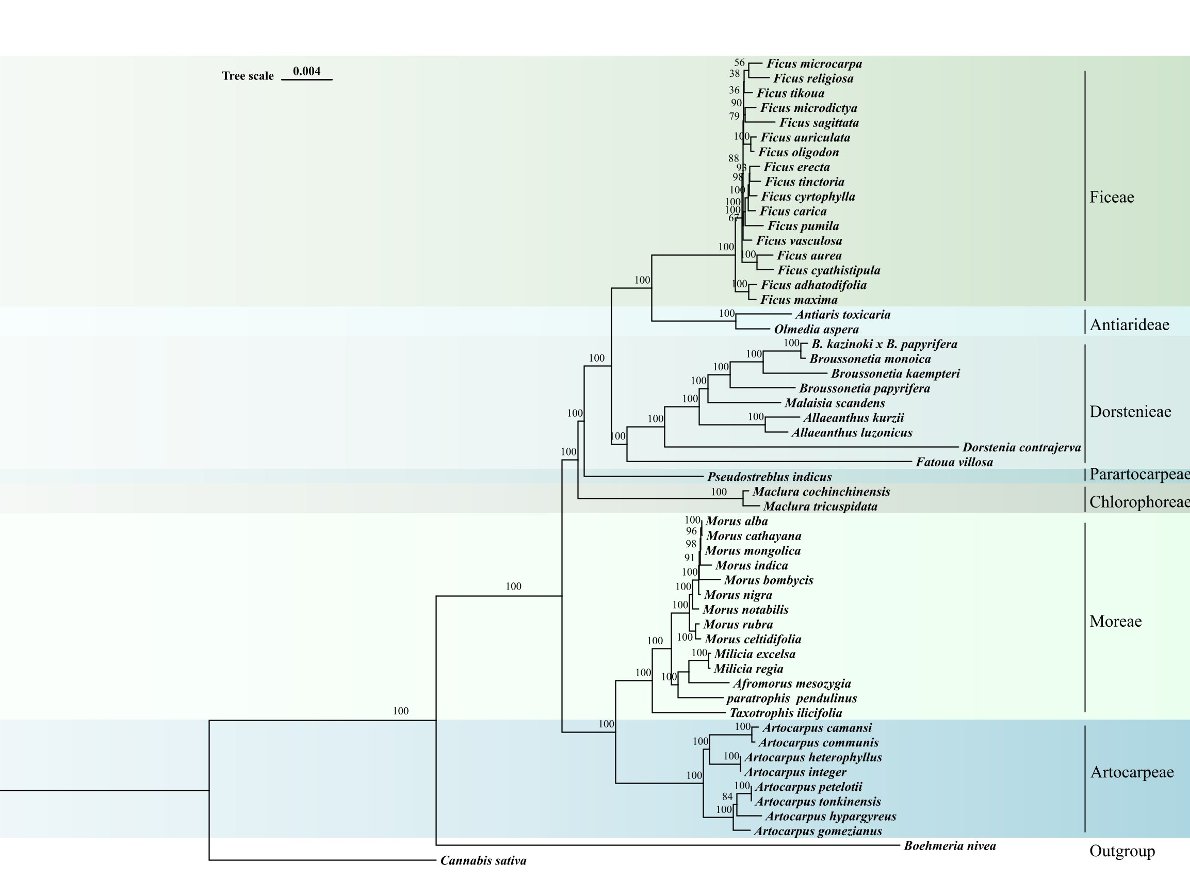


**Figure S8** The phylogenetic tree (ML) constructed based on the protein-coding genes of the plastid genomes of 53 Moraceae plants.


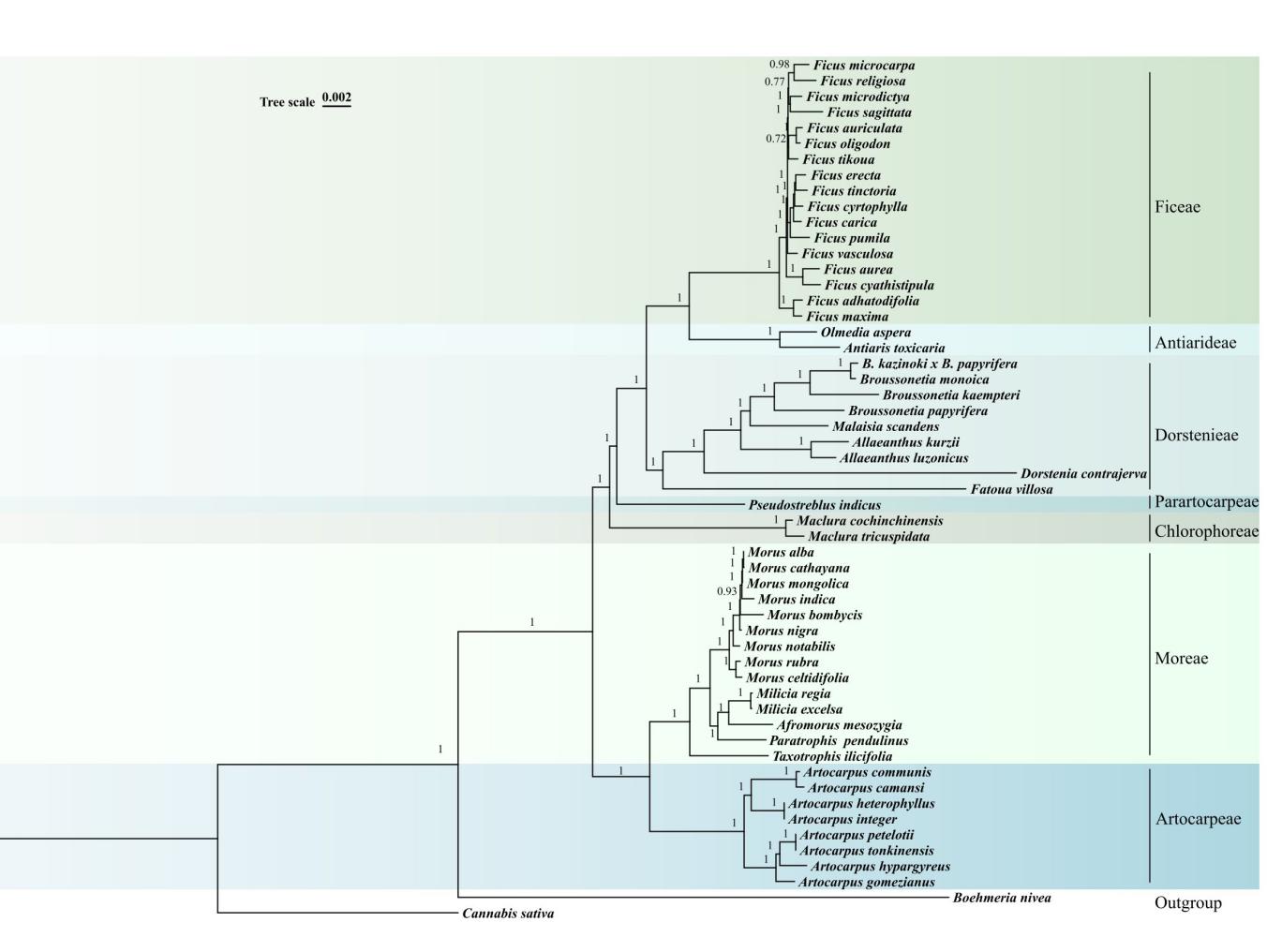


**Figure S9** The phylogenetic tree (Bayes) constructed based on the protein-coding genes of the plastid genomes of 53 Moraceae plants.


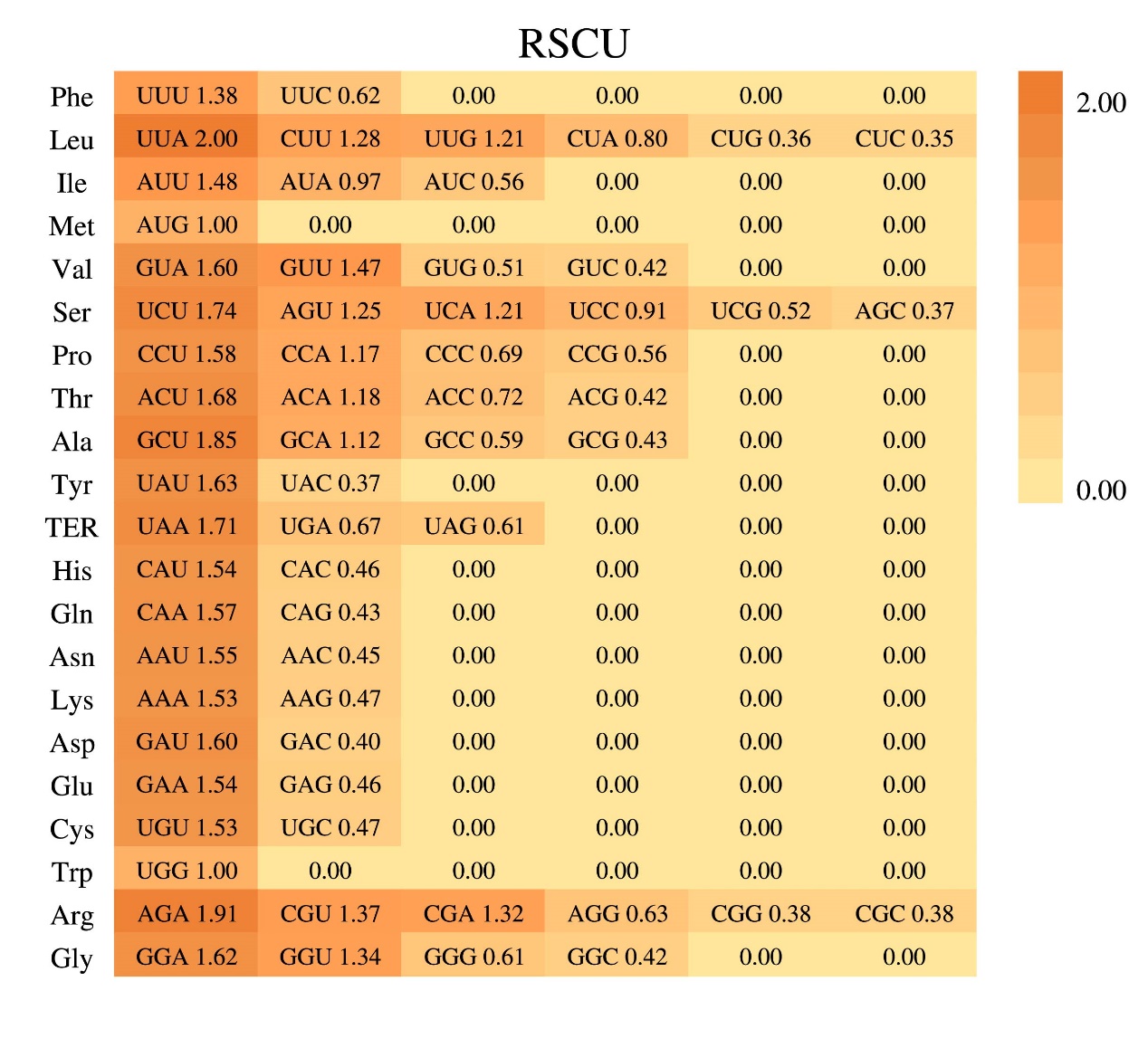
 **Figure S10** Relative Synonymous Codon Usage (RSCU) frequency across 53 Moraceae species.


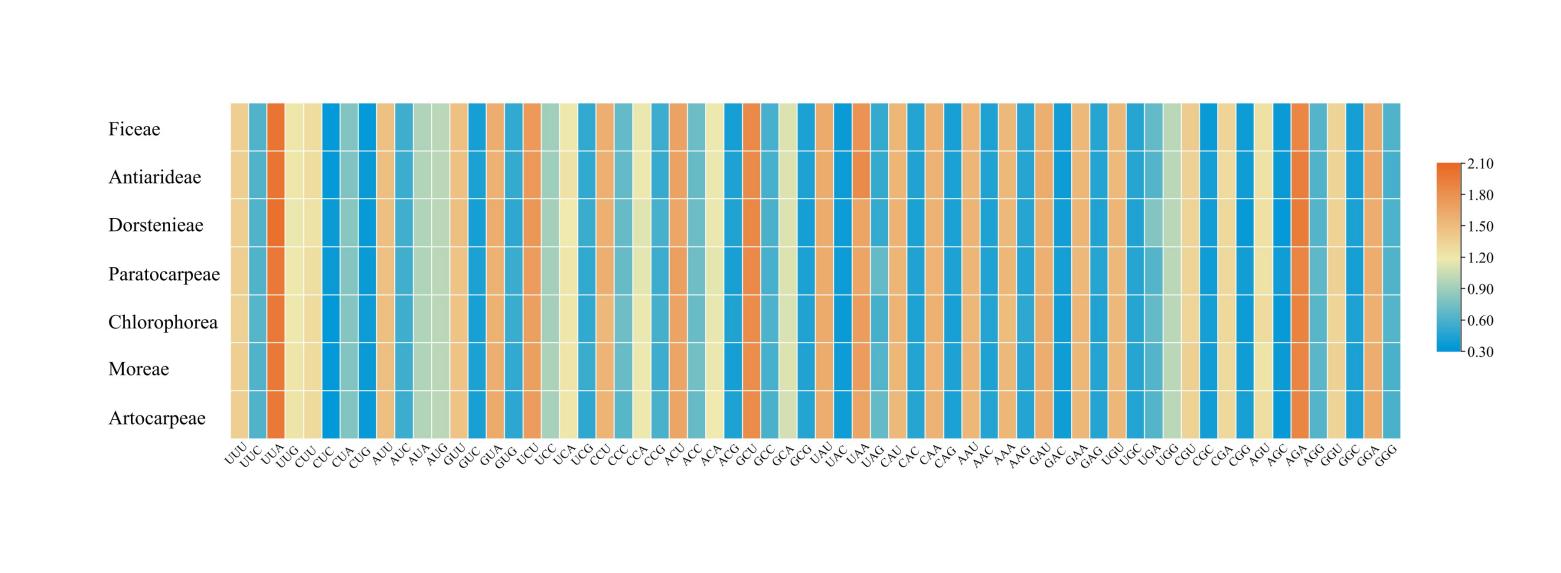


**Figure S11** The codon usage heatmap distribution of different tribes of Moraceae.


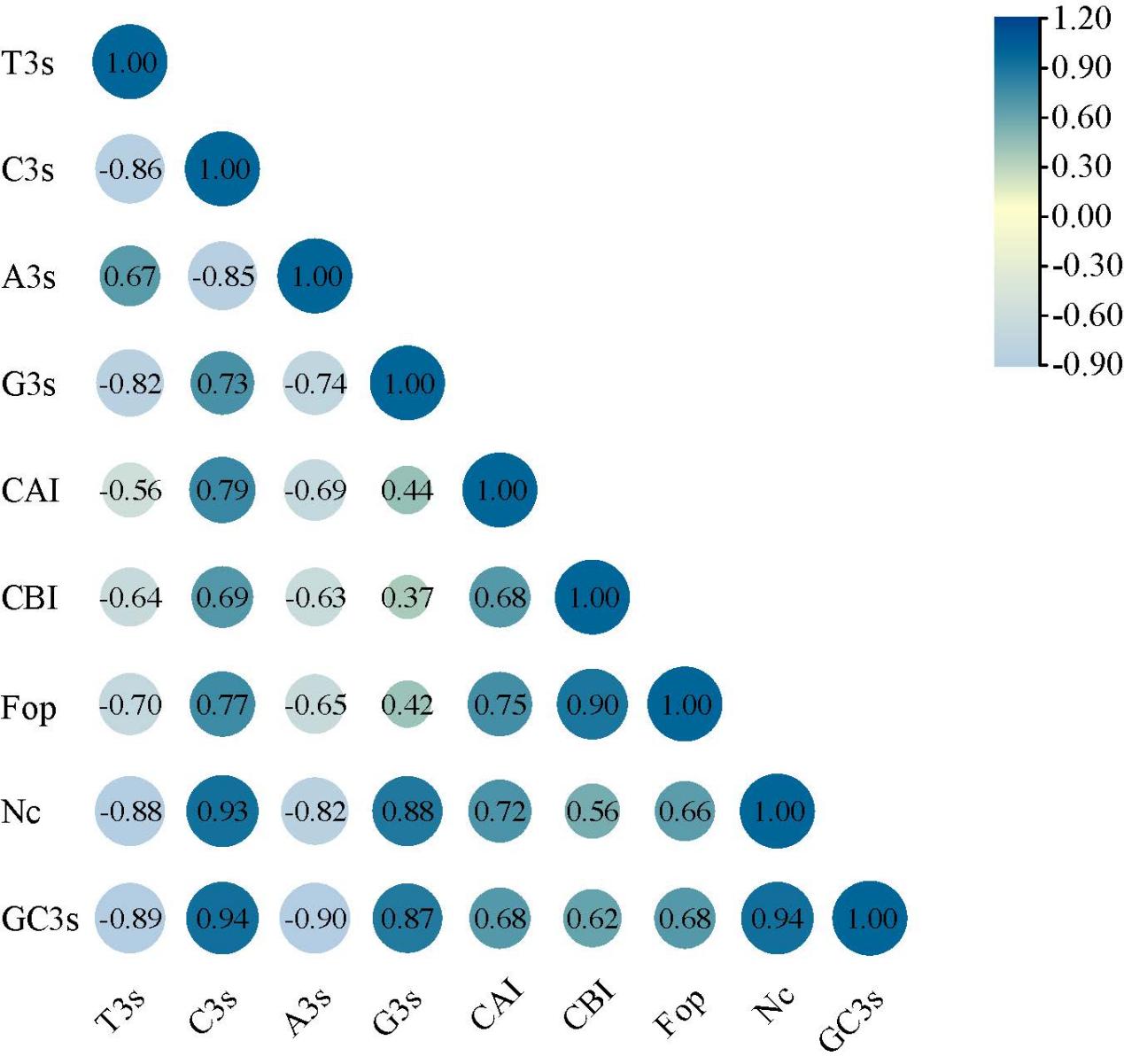


**Figure S12** Heatmap of gene correlation coefficients among plastomes of Moraceae species.


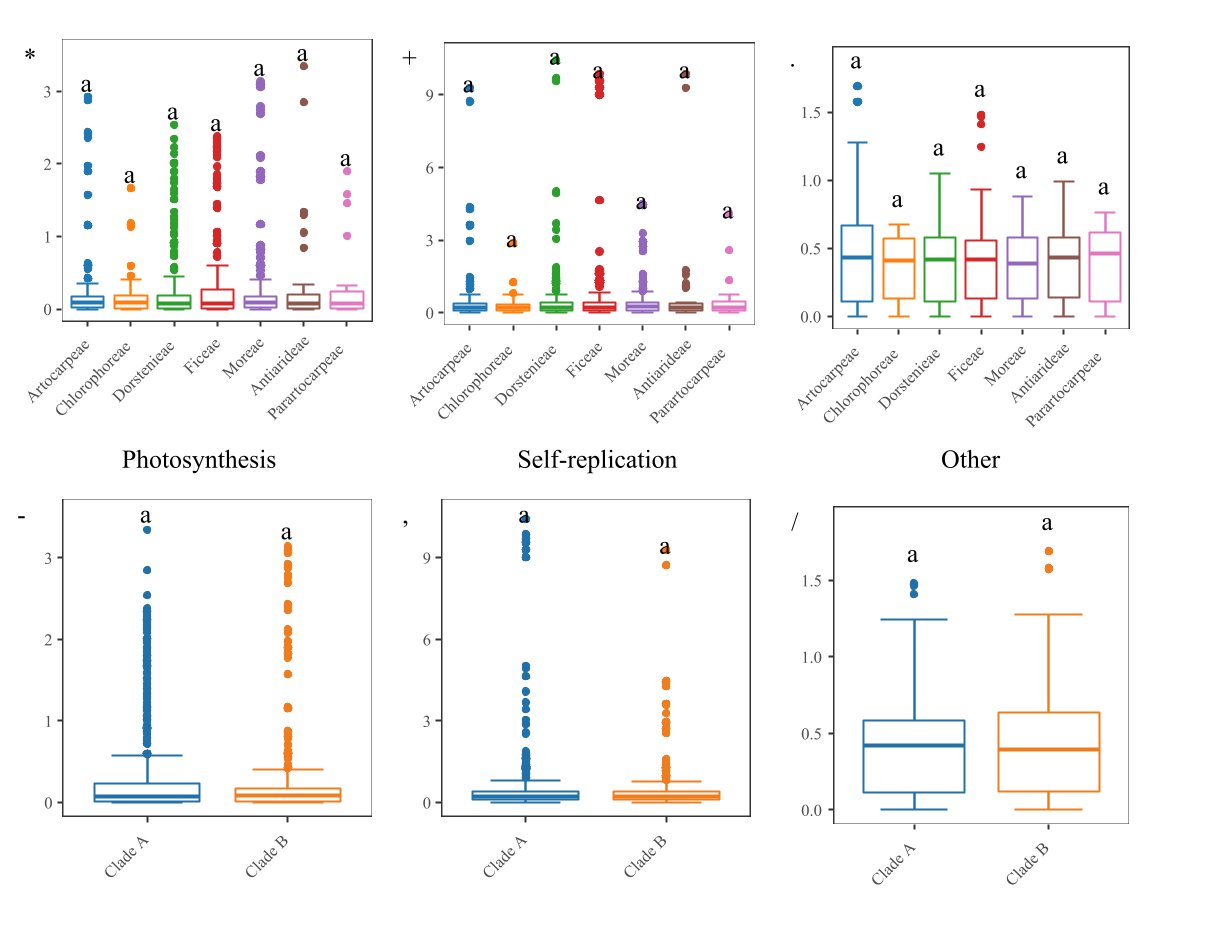


**Figure S13** Functional classification gene selection pressure analysis for different tribes and clades of Moraceae. **(A)** Gene selection pressure analysis for photosynthesis-related genes in different tribes of Moraceae. **(B)** Gene selection pressure analysis for self-replication-related genes in different tribes of Moraceae. **(C)** Gene selection pressure analysis for other functional genes in different tribes of Moraceae. **(D)** Gene selection pressure analysis for photosynthesis-related genes in different clades of Moraceae. **(E)** Gene selection pressure analysis for self-replication-related genes in different clades of Moraceae. **(F)** Gene selection pressure analysis for other functional genes in different clades of Moraceae.
